# The BEACH Protein LRBA Promotes the Localization of the Heterotrimeric G-protein G_olf_ to Olfactory Cilia

**DOI:** 10.1038/s41598-017-08543-4

**Published:** 2017-08-16

**Authors:** Stefan Kurtenbach, Andreas Gießl, Siv Strömberg, Jan Kremers, Jenny Atorf, Sebastian Rasche, Eva M. Neuhaus, Denis Hervé, Johann Helmut Brandstätter, Esther Asan, Hanns Hatt, Manfred W. Kilimann

**Affiliations:** 1 Department of Cell Physiology, Ruhr University Bochum, D-44780 Bochum, Germany; 2 Department of Biology, Animal Physiology, University of Erlangen-Nürnberg, D-91058 Erlangen, Germany; 3 Department of Neuroscience, Uppsala University, S-75124 Uppsala, Sweden; 4 Department of Ophthalmology, University Hospital Erlangen, D-91054 Erlangen, Germany; 5 Department of Anatomy II, Friedrich-Alexander University Erlangen-Nürnberg, D-91054 Erlangen, Germany; 6 Department of Pharmacology and Toxikology, University Hospital Jena, D-07747 Jena, Germany; 7 Inserm UMR-S839, Institut du Fer a Moulin, Universite Pierre et Marie Curie, F-75005 Paris, France; 8 Institute of Anatomy and Cell Biology, University of Würzburg, D-97070 Würzburg, Germany; 9 Department of Molecular Neurobiology, Max Planck Institute for Experimental Medicine, D-37075 Göttingen, Germany

## Abstract

BEACH domain proteins are involved in membrane protein traffic and human diseases, but their molecular mechanisms are not understood. The BEACH protein LRBA has been implicated in immune response and cell proliferation, and human LRBA mutations cause severe immune deficiency. Here, we report a first functional and molecular phenotype outside the immune system of LRBA-knockout mice: compromised olfaction, manifesting in reduced electro-olfactogram response amplitude, impaired food-finding efficiency, and smaller olfactory bulbs. LRBA is prominently expressed in olfactory and vomeronasal chemosensory neurons of wild-type mice. Olfactory impairment in the LRBA-KO is explained by markedly reduced concentrations (20–40% of wild-type levels) of all three subunits α_olf_, β_1_ and γ_13_ of the olfactory heterotrimeric G-protein, G_olf_, in the sensory cilia of olfactory neurons. In contrast, cilia morphology and the concentrations of many other proteins of olfactory cilia are not or only slightly affected. LRBA is also highly expressed in photoreceptor cells, another cell type with a specialized sensory cilium and heterotrimeric G-protein-based signalling; however, visual function appeared unimpaired by the LRBA-KO. To our knowledge, this is the first observation that a BEACH protein is required for the efficient subcellular localization of a lipid-anchored protein, and of a ciliary protein.

## Introduction

The BEACH domain is the defining feature of a protein family which expanded from a single progenitor in yeasts to 4–9 members in multicellular organisms as diverse as *Dictyostelium*, *C*. *elegans*, *Drosophila*, plants and vertebrates. Most BEACH proteins are large (~3000 amino acids [aa]) and share a domain architecture in which the highly conserved BEACH domain (~280 aa) is followed by a WD repeat at the C-terminus, whereas the N-terminal sequence regions are more divergent. Since the identification of this protein family in 1997, little has been learned about how they function at the molecular level. Deficiency mutants of various BEACH proteins were characterized in humans, mice, flies, worms, slime molds, plants and yeast, displaying complex phenotypes of various degrees of severity. As a common denominator, a role in the dynamics of endomembranes (often involving endosomes, lysosomes/vacuoles, autophagosomes and secretory granules) and of transmembrane proteins seems to be emerging.

Several BEACH proteins are implicated in human diseases^1^. LYST (lysosomal trafficking regulator) is mutated in Chédiak-Higashi syndrome and the *beige* mouse (the acronym BEACH is derived from “beige and Chédiak-Higashi”). LYST deficiency gives rise to giant lysosomes and perturbations in the biogenesis of lysosome-derived secretory granules, resulting in defects of pigmentation, thrombocyte function, immune response and neurological functions. Mutations in NBEAL2 (Neurobeachin-like protein 2) cause Gray Platelet Syndrome, with abnormalities in the biogenesis of thrombocytes and their secretory α-granules. Mutations in WDR81 or WDFY3 underlie severe neurodevelopmental defects in humans and mice. Heterozygous NBEA (Neurobeachin) gene rearrangements have been detected in groups of patients with either autism or monoclonal gammopathy and multiple myeloma. Moreover, reduced NBEA expression causes overweight in mice and may also be involved in human obesity^2^.

LRBA (LPS-responsive beige-like anchor protein) and NBEA are each other’s closest relatives within the BEACH protein family. Whereas NBEA is prominently expressed in neurons and endocrine cells and has a high-affinity binding site for protein kinase A (PKA)^3^, LRBA is expressed in many tissues and cell types^4^ and does not seem to bind PKA^3^. NBEA and LRBA have diverged only in vertebrates^5^. *C*. *elegans* and *Drosophila* have a single progenitor which can bind PKA (in *Drosophila* at least) and whose deficiency gives rise to moderate defects of morphogenesis and growth factor receptor function^6–8^. LRBA was identified as a gene product which is up-regulated in stimulated immune cells and in cancer cells^4, 9^. Consistent with these experimental findings, genetic LRBA deficiency causes immunological abnormalities in humans^10–13^ and mice^14^. Emerging evidence also implicates LRBA in breast cancer^9, 15^.

In the present study, we set out to explore the biological role of LRBA by generating LRBA knockout (KO) mice. These mice are viable and fertile, but the assays of the phenotyping screen carried out by the German Mouse Clinic (www.mouseclinic.de/screens/immunology) detected no perturbed immune functions. Instead, upon closer scrutiny we found impaired olfaction by LRBA-KO mice, concurrent with reduced abundance of the heterotrimeric G-protein, G_olf_, in the sensory cilia of olfactory neurons. With these results, BEACH proteins continue to emerge as a novel and scarcely explored molecular principle in the orchestration of subcellular protein distribution.

## Results

### Tissue distribution of LRBA expression, and construction of LRBA gene-modified mice

**Figure 1:**
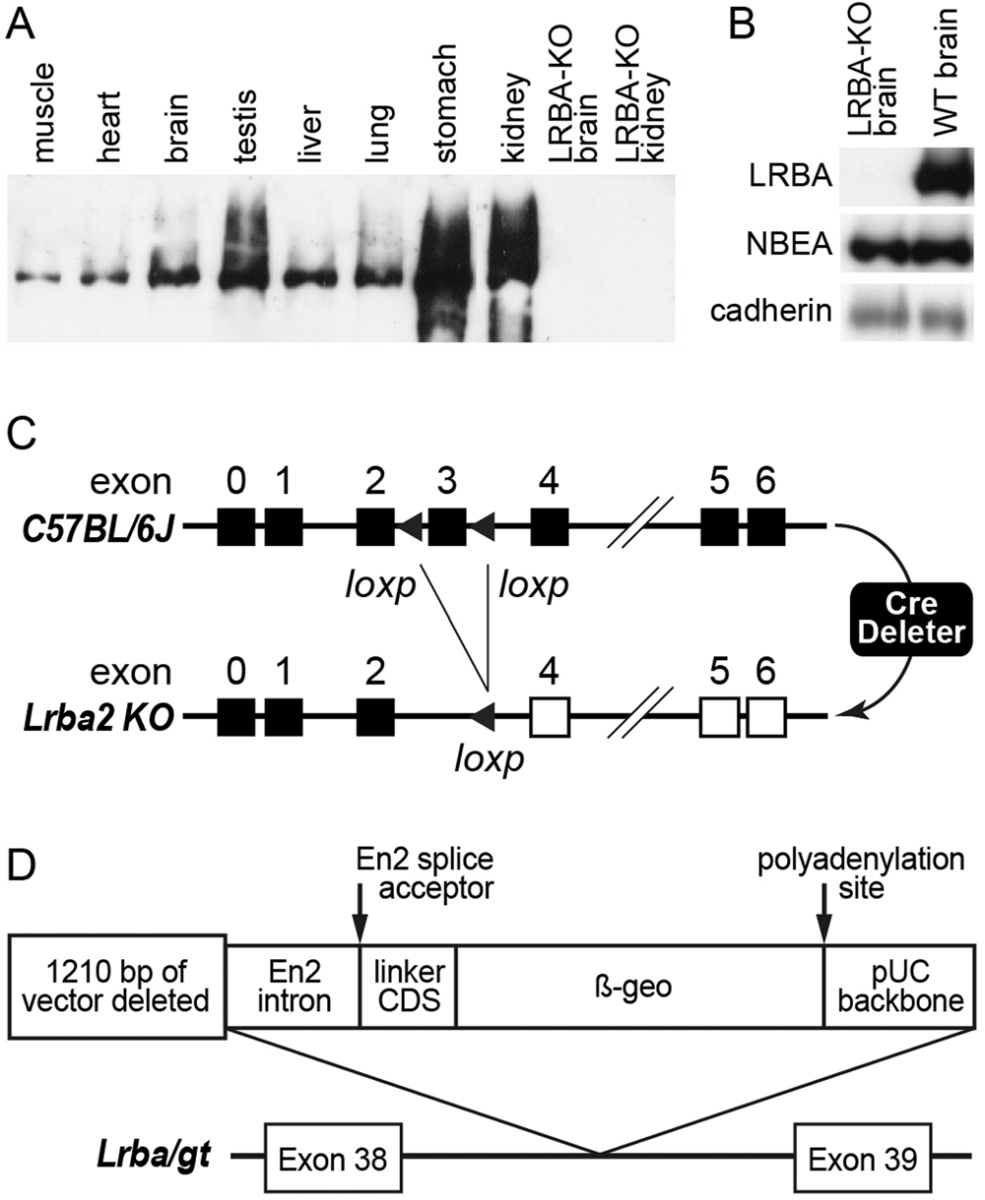
LRBA expression in WT and KO mouse tissues; *Lrba* gene modifications. (**A**) LRBA protein (~320 kDa) is detected by immunoblotting in all WT tissues tested, but is undetectable in KO mouse tissues. An unprocessed image of this immunoblot is shown in Supplementary Fig. S1. (**B**) Immunoblots of brain homogenates from LRBA-KO and WT mice were sequentially developed with anti-LRBA, anti-NBEA, and anti-pan-cadherin as control; the image shows slivers with the bands representing these three proteins. No cross-reaction with NBEA is detectable in LRBA-KO brain, confirming the specificity of the LRBA antibody in immunoblotting. Loading, 20 µg protein/lane. (**C**) Mutation strategy of the *Lrba2* KO. The 5′-terminal noncoding exon is termed exon 0. (**D**) Gene-trap constellation in the hypomorphic *Lrba/gt* mutant mice expressing β-galactosidase reporter enzyme activity.

We raised antisera against a region of the mouse LRBA sequence not conserved in NBEA or other BEACH proteins. Immunoblot analysis of wild-type (WT) mouse tissues detected a protein band of the expected size (~320 kDa) in all tissues tested, most abundantly in stomach and kidney (Fig. 1A).

We produced two lines of LRBA-mutant mice. Targeted deletion of coding exon 3 generated a frameshift after 5% of the coding sequence (149 aa), giving rise to a constitutive KO (laboratory allele designation, *Lrba2*) (Fig. 1C, Methods). Immunoblot analysis showed that this mutation rendered LRBA undetectable in brain and kidney homogenates (Fig. 1A,B), even with much longer exposures than in Fig. 1 (detection limit, ~1% of WT expression). Formally, it remains possible that proteins representing either the N-terminal 149 aa alone, or spliced in-frame to coding exons downstream of the immunogen sequence (which largely corresponds to coding exon 22) and thus undetectable by the antibody, remain stably expressed and exert hypomorphic or dominant-negative effects, but this seems unlikely. We also performed RT-PCR on RNA from *Lrba2* and WT mouse brain between coding exons 1 and 5, and obtained intense, clean, single bands from both, the band from *Lrba2* being appropriately shorter. Sequencing verified the intact LRBA cDNA sequence covered by this primer pair in the WT PCR product, whereas the coding exon 3 sequence interval was cleanly deleted from the *Lrba2* cDNA. These data demonstrate that the mutation functioned as intended, that no physiological differential splicing occurs in WT brain between coding exons 1–5, and that no cryptic splice sites within this interval were activated by the deletion of coding exon 3 which might have circumvented the frameshift. Therefore, we deem it highly likely that *Lrba2* is a null mutation, and refer to it as LRBA-KO from now on. No compensatory up-regulation of NBEA was observed in the brain of LRBA-KO mice (Fig. 1B). Homozygous mutant mice were viable and fertile, displaying no obvious abnormalities or increased mortality up to the age of 2 years, similar to the independent LRBA-KO mice of ref. 14.

Blastocyst injection of a gene-trap ES cell line produced a mouse mutant (laboratory allele designation, *Lrba/gt*) predicted to express a fusion protein of the N-terminal 71% of the LRBA coding sequence (2015 aa) with β-galactosidase, under the control of the endogenous *Lrba* gene promoter (Fig. 1D). Immunoblot analysis of brain and kidney with anti-LRBA revealed that this gene-trap was leaky, with homozygotes still expressing ~25% of the normal-sized LRBA protein. However, the *Lrba/gt* mouse line allowed the visualization of the histological expression pattern of LRBA by LacZ enzyme histochemistry.

### LRBA-KO mice have impaired olfaction

**Figure 2:**
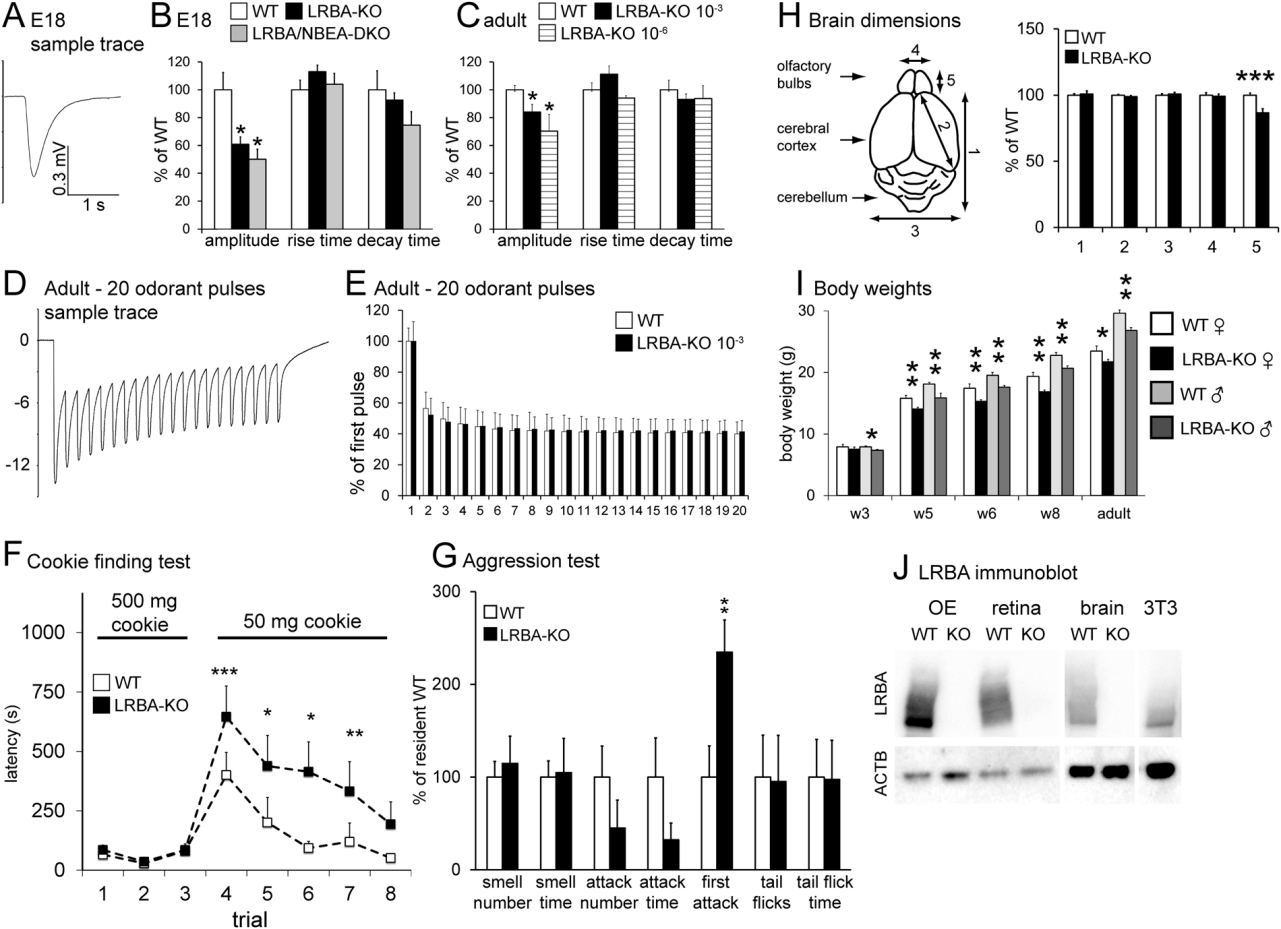
Impaired olfaction of LRBA-KO mice indicated by OE electrophysiology, behaviour, and reduced olfactory bulb size and body weight. (**A**) EOG sample trace (H100 at 10^−3^ for 100 ms) of a WT mouse embryo (e18). (**B**) H100-elicited EOG amplitudes from e18 LRBA-KO mice (n = 85 OE specimens from 13 animals) were reduced to 60% of WT controls (n = 35, 7 animals). LRBA/NBEA-DKO did not significantly reduce the amplitudes further (n = 34, 7 animals). Signal rise and decay kinetics were unaffected. Mean WT values were: amplitude, 0.62 mV; rise time, 0.14 s; decay time, 1.14 s. *p < 0.05 relative to WT in parts B and C (**C**) Adult LRBA-KO animals also had reduced EOG amplitudes (by 16%) (WT: n = 198, 7 animals; LRBA-KO: n = 176, 7 animals) and unaltered signal kinetics (mean WT amplitude, 5.44 mV; rise time, 0.10 s; decay time, 0.89 s). At low odor concentrations (H100 10^−6^; in all other EOG measurements, 10^−3^), amplitudes were reduced by 30% (WT: n = 62, 6 animals; LRBA-KO: n = 45, 5 animals) (mean WT amplitude, 1.02 mV; rise time, 0.13 s; decay time, 0.73 s). (**D**,**E**) Adaptation kinetics (20 H100 odor pulses, 1 s application, 4 s interval, n = 5 adult animals, one measurement per animal) was unaffected by the LRBA-KO. (**F**) The cookie-finding test was performed on 8 consecutive days. Under the more difficult conditions (smaller cookie), LRBA-KO animals (n = 10) needed significantly more time to find the cookie than WT mice (n = 13). (**G**) Male aggression in the resident-intruder paradigm was reduced by the LRBA-KO (n = 9 WT and KO residents each). (**H**) LRBA-KO mice (n = 7; age 14d) showed a reduced olfactory bulb length (parameter 5); other dimensions of the brain did not differ from age-matched WT controls (n = 13). (**I**) Body weight of LRBA-KO animals vs. littermate WT controls was reduced, regardless of sex and age (3–16 weeks). Animal numbers: WT females, 5–6; WT males, 9–10; KO females, 11–14; KO males, 6–8. (**J**) LRBA protein is expressed in WT mouse OE (20 µg protein/lane), retina, brain and 3T3 cells (each, 50 µg), and abolished in the KO mouse tissues. LRBA immunoblots display the MW range from 250 kDa upwards to top of gel. β-actin signals (ACTB) as loading controls are shown below.

As NBEA and LRBA are both implicated in the subcellular distribution of membrane proteins, and a survey of multiple *Lrba/gt* mouse tissues by whole-mount LacZ staining indicated LRBA expression in olfactory epithelium (OE), we investigated their involvement in the functional expression of proteins of the olfactory signal transduction chain. First, we performed air-phase electro-olfactograms (EOG) of the OE (Fig. 2A–E). We compared the results from embryonic day 18 (e18) mice, deficient either for LRBA alone, or additionally for NBEA^16^ (LRBA/NBEA-DKO), with those of age-matched WT controls, applying a mixture of 100 different odorants (Henkel 100 [H100], 1:1000). Late-embryonic animals were initially used because the LRBA/NBEA-DKO, like the NBEA-KO^16, 17^, is neonatal-lethal due to breathing paralysis. Figure 2B shows that response amplitudes of e18 LRBA-KO mice were reduced to 60%, whereas the additional ablation of NBEA caused no significant further reduction of the EOG response. The kinetics of the individual EOG signals (rise and decay times) were unaffected, indicating no major effects on the mechanisms of signal amplification or termination. As LRBA-KO mice are viable, unlike NBEA-KO mice, we subsequently examined adult mice (Fig. 2C) and detected similar though apparently weaker response amplitude reductions at high (10^−3^) and low (10^−6^) H100 odor concentrations. Repetitive odorant stimulation (Fig. 2D,E) revealed no difference in the desensitization kinetics between LRBA-KO and WT mice, giving no indication of effects on adaptation mechanisms such as the phosphorylation of olfactory receptors (ORs).

**Figure 3:**
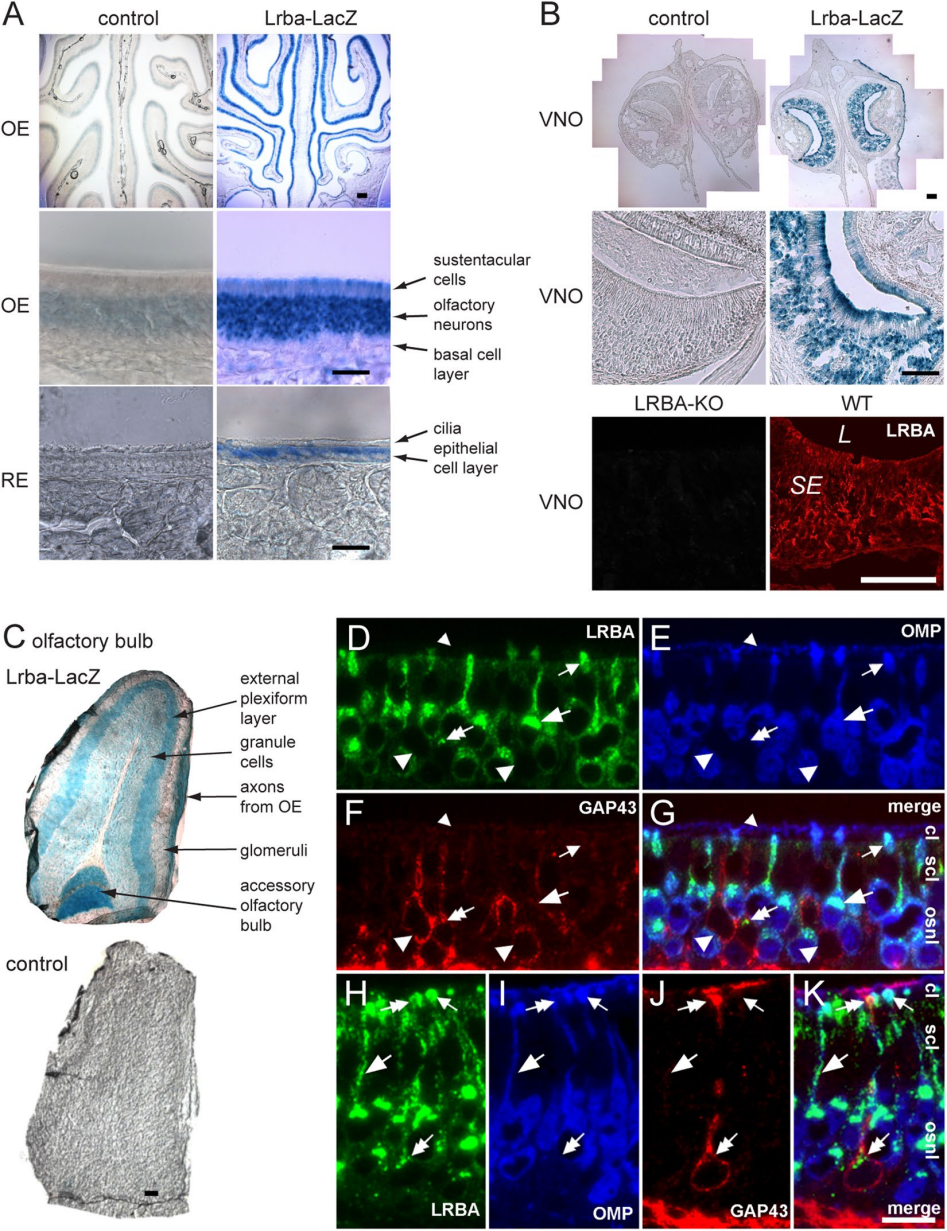
Histological and subcellular distribution of LRBA in olfactory and vomeronasal tissues. (**A**) LacZ enzyme histochemistry of *Lrba/gt* gene-trap mice (age, 3 weeks) demonstrates expression of the LRBA gene throughout the OE (top). Larger magnification (middle) shows that the staining is predominantly localized to the OSNs. Respiratory epithelium (RE, bottom) is also stained, though weaker than OE. (**B**) β-galactosidase staining was also seen in VNO neuron somata and the microvillar layer of *Lrba/gt* mice (top, middle), and confirmed by LRBA-IF of adult WT tissue (bottom; SE, sensory epithelium; L, lumen). Scale bars in **A**,**B**: overviews 100 µm, close-ups 25 µm. (**C**) LacZ staining of the olfactory bulb required longer incubation (16 h) than of the OE and VNO (2 h). Scale bar in **C**: 100 µm. (**D**–**K**) Triple immunostaining for LRBA (**D**,**H**), OMP (**E**,**I**), GAP43 (**F**,**J**) and merged images (**G**,**K**) in different regions of the adult mouse OE of low (**D**–**G**) or high (**H–K**) thickness. LRBA-immunoreactivity (ir) is found in the apical somatic cytoplasm and in dendrites as well as dendritic knobs of virtually all OMP-ir mature OSNs. Large and small arrows point to select LRBA-/OMP-ir cell bodies/dendrites and dendritic knobs, respectively, but LRBA is lacking in the OMP-ir ciliary layer (small arrowheads in **D**–**G**). GAP43-ir immature neurons contain no (large arrowheads in **D**–**G**) or only small amounts (double arrows in **D**–**K**) of LRBA-immunoreactivity in the apical cytoplasm of their cell bodies and in dendritic knobs. Scale bar in **K** for **D**–**K**: 10 µm. OE strata are designated in panels G and K: cl, cilial layer; scl, sustentacular cell layer; osnl, OSN layer.

The behaviour of LRBA-KO mice also indicated olfactory impairment. In a cookie-finding test with easy settings (large cookie, 500 mg), LRBA-KO and WT mice performed equally well (Fig. 2F, trials 1–3). When test conditions were more difficult (small cookie, 50 mg), LRBA-KO mice did not perform as well as control animals (Fig. 2F, trials 4–8). Performances of both mouse groups improved during repeated trials (trials 4–8) as the mice learned. As the learning curves were similar for both genotypes, LRBA-KO mice did not appear to have major impairments of learning or memory under these conditions. As mouse social behaviour is critically influenced by olfactory and pheromonal cues, and LRBA is prominently expressed in the vomeronasal organ (VNO) and the accessory olfactory bulb to which VNO sensory neurons project (see Fig. 3, below), we also performed male aggression tests. We observed a significant delay of the resident males’ first attack, and tendentially reduced number and total time of attacks (Fig. 2G).

LRBA deficiency also affected the macroscopic morphology of the mice in ways consistent with impaired olfaction. Olfactory bulb size was reduced (Fig. 2H), as in other hypoosmic mouse mutants^18^ or under olfactory deprivation by naris occlusion^19^. LRBA-KO mice had reduced body weight (Fig. 2I), continuously observable from juvenile to adult age and in males and females alike. Analysis of several additional mouse cohorts always confirmed the reduced body weight of the LRBA-KO mice, also at younger ages (1 and 2 weeks). Reduced weight may be due to impaired olfactory feeding appeal as in other olfaction-impaired mice^18–21^, but also e.g. to dysfunctions of the kidney or the gastrointestinal tract (organs in which LRBA is highly expressed, see Fig. 1A), or to a generalized trophic impairment^9^.

Immunoblot analysis confirmed that LRBA is expressed in OE, and also in retina, and that the KO abolishes expression of the protein also in these tissues (Fig. 2J). Comparison with the brain signal intensity shows that OE and retina are highly LRBA-expressing tissues, similar to kidney and stomach (Fig. 1A). LRBA was also detected in mouse cell lines 3T3 and IMCD3.

### LRBA is expressed in olfactory and vomeronasal sensory neurons

To examine the histological LRBA expression pattern, we first performed β-galactosidase enzyme histochemistry of *Lrba/gt* gene-trap mouse tissue slices, which express β-galactosidase activity under the control of the *Lrba* gene promoter. Marked *Lrba* reporter expression was detected throughout the OE area (Fig. 3A, top), primarily in the olfactory sensory neurons (OSN) (Fig. 3A, middle). Also the respiratory epithelium was found to express the *Lrba* reporter (Fig. 3A, bottom), like many other epithelial and neuronal cell types of the mouse. In the VNO, expression was seen in both apical and basal layers of sensory neurons as well as in the non-sensory epithelium of the opposing convex luminal wall (Fig. 3B). In the olfactory bulb (Fig. 3C), the layer of axons from the OSNs, the external plexiform layer, and both the anterior and posterior parts of the accessory olfactory bulb (to which the axons from the apical and basal vomeronasal sensory neurons respectively project) were intensely stained. The glomerular and granule cell layers displayed weaker staining.

LacZ histochemistry has the advantage of high sensitivity but limited spatial resolution. The LacZ fusion protein typically distributes throughout the cytosol of the OSNs and therefore yields no information on the subcellular localization of native LRBA, and the color reaction product tends to bleed across cell borders. Therefore, we next performed triple-immunofluorescence (IF) labeling of semithin sections from freeze-dried, epon-embedded WT OE (Fig. 3D–K) with anti-LRBA, anti-OMP (a marker for mature OSNs) and anti-GAP43 (a marker for immature OSNs). This detected LRBA in the cytoplasmic space of OSNs, clustering at the apical cell poles of somata and in the dendrites (large arrows in Fig. 3D–K) and dendritic knobs (small arrows) from which the sensory cilia emanate. The layer of the olfactory cilia proper was OMP-immunoreactive (ir) but LRBA-negative (small arrowheads in Fig. 3D–G). At high magnification, LRBA subcellular localization appeared granular or patchy, similar to NBEA^3^. Analysis of various OE regions revealed LRBA localization in apparently all OMP-ir OSNs (Fig. 3D,E,G and H,I,K), whereas most GAP43-ir immature neurons displayed no (large arrowheads) or else much sparser LRBA labeling (double arrows) in their apical cytoplasm (Fig. 3D,F,G). Only rarely, LRBA immunoreactivity extended into the dendrite and/or dendritic knob of GAP43-ir neurons (Fig. 3H,J,K). This indicates that LRBA expression in OSNs is up-regulated synchronously with functional maturation, i.e. with the onset of olfactory cilia formation and signalling^22^. Sustentacular cell bodies surrounding the OSN dendrites in the apical epithelium were much more sparsely labeled for LRBA than the OSNs.

In the VNO (Fig. 3B, bottom), IF of cryosections stained both sensory neuron strata up to their microvillar layer, in agreement with the LacZ histochemical staining above. IF also confirmed the LRBA expression in respiratory epithelium (not shown), as demonstrated in Fig. 3A (bottom) by LacZ histochemistry. As shown here for the VNO (Fig. 3B, bottom), LRBA IF was likewise abolished in the OE of LRBA-KO mice (not shown), confirming the specificity of staining.

### Ciliary abundances of all three G_olf_ subunits are reduced in LRBA-KO mice

As BEACH proteins are thought to be involved in the subcellular targeting of membrane proteins, we suspected that LRBA deficiency reduces the EOG signal through an impact on proteins of the olfactory signal cascade. The electrophysiological and behavioural impairments of olfaction in the LRBA-KO mice were modest, but olfactory signalling proteins seemed a particularly attractive subject to investigate their potential mistargeting because of their marked concentration in the olfactory cilia. Moreover, KO mice for some olfactory signalling proteins show that even the total absence of proteins considered crucial for olfaction^23, 24^ may result in only mild or undetectable effects on commonly used olfactory test parameters. We therefore felt encouraged to probe for the expression and localization of numerous olfactory signalling proteins in OE of LRBA-KO and WT mice.

**Figure 4:**
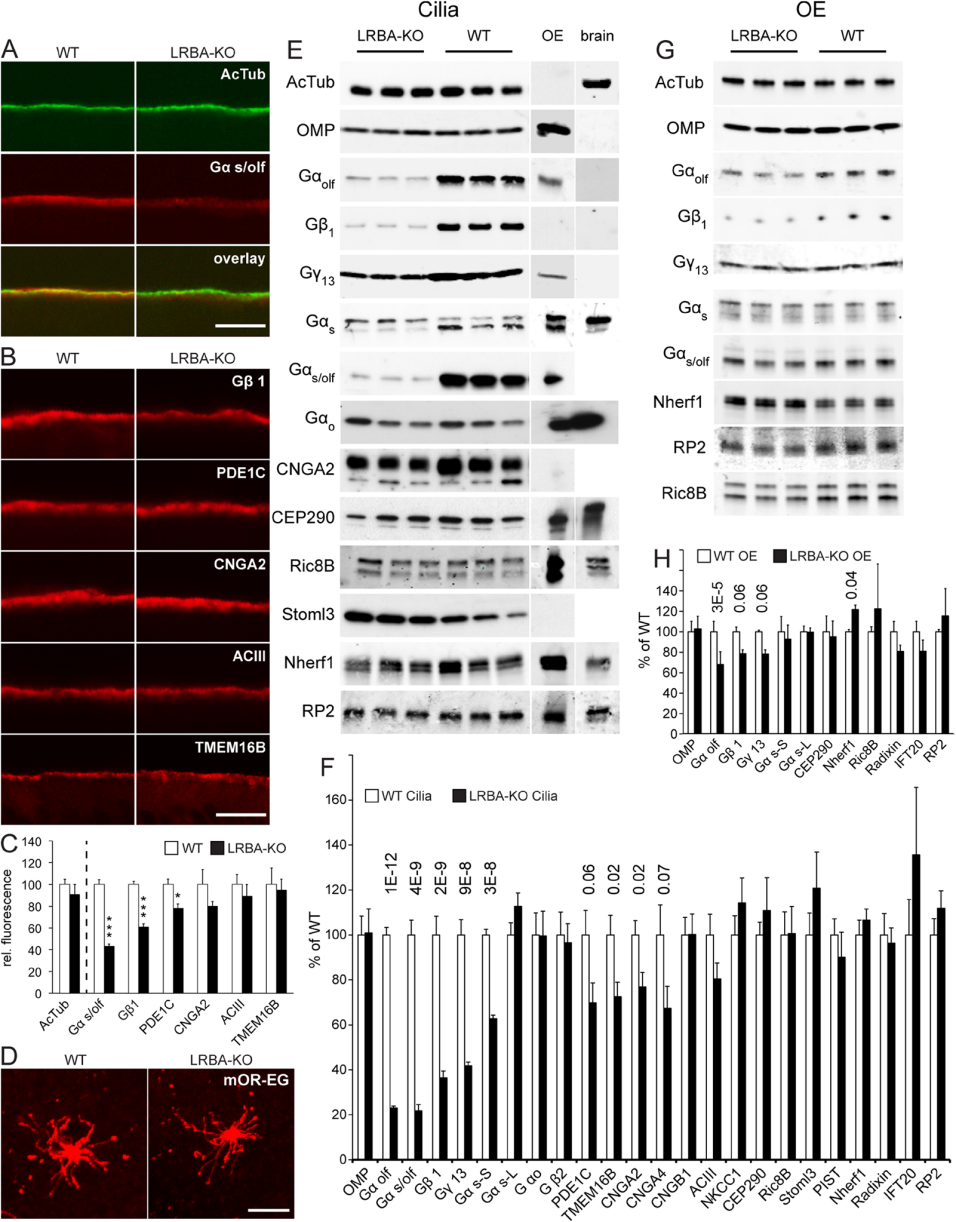
Reduced abundances of G_olf_ subunits in olfactory cilia of LRBA-KO mice. (**A**) Double staining of an OE cross-section for acetyl-tubulin (green) and Gα_s/olf_ (red). (**B**) Representative stainings of several ciliary proteins in OE cross-sections of WT and LRBA-KO mice aged 14d. (**C**) Quantification of fluorescence intensities. Values for acetyl-tubulin in KO and WT specimens, set apart by a vertical dashed line, are shown in direct comparison, whereas values for all other antigens were normalized on the respective KO and WT acetyl-tubulin values. (**D**) En-face views of whole mount stainings of ciliary tufts with an antibody specific for the olfactory receptor mOR-EG (Olfr-73). Scale bars, 7 µm. (**E**) Representative examples of immunoblot images of purified olfactory cilia. Three distinct cilia preparations each from LRBA-KO and WT mice (age, 4 weeks) were blotted in parallel (10 µg protein each), along with an OE and a brain sample (50 µg protein each), all shown at the same exposure. (**F**) Quantitative evaluation of cilia immunoblot analysis, normalized on acetyl-tubulin signal strength. p-Values <0.1 are given above the columns of the respective proteins; n = 9 for most antigens except PDE1C, CEP290 (n = 6), and Gα_olf_ (n = 12). Note that the bar diagrams F and H contain quantification results of more proteins than the example images of parts **E**+**G**. (**G**) Examples of immunoblot images of three OE samples each from LRBA-KO and WT mice (age, 4 weeks) blotted in parallel. (**H**) Quantitative evaluation of OE immunoblot analysis, normalized on acetyl-tubulin signal strength; n = 6 for most antigens except Gβ_1_, Nherf1, Gγ_13_, IFT20, radixin (n = 3), Ric8B and CEP290 (n = 9). p-Values < 0.1 are given above the columns of the respective proteins. Molecular weights of the bands shown in parts E and G, and/or quantified in parts F and H, are consistent with the predicted molecular sizes and/or the literature. Specificity of the Ric8B double-band was confirmed by a KO control (see Methods).

IF microscopy of OE cross-sections showed the typical, sharply demarcated staining of the ciliary layer for the cilia marker, acetyl-tubulin, and for several proteins of the olfactory signal chain (Fig. 4A,B). These proteins all concentrate in the long sensory cilia at the OE surface, horizontally criss-crossing to form a dense, fleece-like tangle which at this low magnification merges into a continuous narrow stripe in cross-section (see e.g. refs 23–26 for similar images). For quantification, WT and KO cryosections were double-stained for each signal protein and for acetyl-tubulin, and IF intensity normalized to the latter. We found no significant difference, between LRBA-KO and WT mice, in the abundance of acetyl-tubulin and of the ciliary transmembrane proteins CNGA2, ACIII, TMEM16B, and a slight reduction of phosphodiesterase PDE1C levels. In contrast, the peripherally membrane-associated protein Gα_olf_ was clearly reduced in the ciliary layer to 43%, and also the IF signal intensity of Gβ_1_, the β subunit isoform of G_olf_ in olfactory cilia^25^, was reduced to 65% of the WT level (Fig. 4C).

The normal abundances of acetyl-tubulin, CNGA2, ACIII and TMEM16B imply that the LRBA-KO does not markedly affect the density of OSNs or the number, size or overall functionality of olfactory cilia. In addition, we visualized a subset of OSNs expressing the olfactory receptor protein (OR), mOR-EG (Olfr73), by whole-mount IF microscopy. Representative en-face views of ciliary tufts are shown in Fig. 4D. At least for mOR-EG-positive OSNs at this level of resolution, there were no obvious effects of the LRBA-KO on OSN density, ciliary tuft morphology, or the radial OR distribution along the cilia.

Olfactory cilia can be dissociated from OE by calcium shock and purified by differential centrifugation. To confirm and extend the IF analysis of the impact of the LRBA-KO on the ciliary abundance of olfactory signalling proteins with an independent technique, we carried out a quantitative immunoblot analysis of multiple cilia preparations from KO and WT mice (Fig. 4E,F). Lanes were loaded with equal protein quantities, and the resultant signals for acetyl-tubulin (reflecting the overall length and number of cilia) as well as OMP (reflecting the overall cytosol volume of cilia) were unaffected by the LRBA-KO (Fig. 4E, top). However, the abundances of all three G_olf_ subunits^26, 27^, Gα_olf_, Gβ_1_ and Gγ_13_, were strikingly reduced to 20–40% in the LRBA-KO samples (Fig. 4E,F). The closest structural and functional relative of Gα_olf_, Gα_s_, developmentally precedes Gα_olf_ in embryonal OSNs and occurs in two splice variants, Gα_s_–S and Gα_s_–L. The ciliary abundance of the directly collinear counterpart of Gα_olf_, Gα_s_–S, was apparently reduced to 60%, whereas the long variant (containing an additional exon) was unaffected by the LRBA-KO.

Analyzed as specificity controls, Gα_o_ and Gβ_2_ were unaffected by the LRBA-KO; both gave weaker signals in the olfactory cilia lanes than in the OE lane, and may largely be derived from tissue debris and/or blood cells contaminating the cilia fraction. In the IF and initial immunoblot analyses, we employed an antibody (sc-383) which is directed against a C-terminal sequence conserved between Gα_olf_ and Gα_s_ and therefore detects both isoforms (designated Gα_s/olf_ in Fig. 4). It was demonstrated previously that in OE of adult mice, Gα_olf_ is the vastly predominant isoform^28^, which is confirmed by the present analysis. All other proteins of the olfactory signal chain for which we tested were unaffected in abundance, or only marginally reduced (PDE1C, TMEM16B, CNGA2, CNGA4) (Fig. 4C,F). The sodium/calcium exchanger NCX1, implicated in the Ca^2+^ homeostasis of olfactory cilia, produced a strong band in brain but remained undetectable in cilia or OE.

It can be seen in Fig. 4E that acetyl-tubulin, the G_olf_ subunits, CNGA2, and also Stoml3, were much more abundant in the WT cilia fractions (each, 10 µg protein) compared to the WT OE sample (50 µg protein) on the same blot and after the same exposure, indicating a marked enrichment in the cilia fraction over total OE. CEP290, Nherf1 or RP2 gave similar signal strengths with cilia and OE, indicative of modest enrichment in cilia, whereas the control protein Gα_0_ gave weaker signals in the cilia samples. Proteins particularly highly enriched in cilia over OE (acetyl-tubulin, Gβ_1_, CNGA2, Stoml3) gave strong cilia signals already at exposures insufficient to produce signals in the OE or brain lanes. To obtain their OE signals, as in part G, longer exposures or higher antibody concentrations were required.

When analyzing total OE protein samples from LRBA-KO and WT in comparison, we again found that both acetyl-tubulin and OMP content (normalized on total protein) were unaffected by the LRBA-KO (Fig. 4G,H). This corroborates further that cumulative cilia length/volume and OSN cell numbers are unaffected by the LRBA-KO. The only proteins whose abundances were significantly or marginally reduced were again Gα_olf_, Gβ_1_ and Gγ_13_ (Fig. 4H), but much less so in OE than in cilia. This suggests that the primary impact of the LRBA-KO is on the enrichment of G_olf_ in the cilia, whereas the slightly reduced total abundance G_olf_ in OE is probably secondary to degradation of mislocalized G_olf_, rather than being a primary, more direct effect of LRBA deficiency on the biosynthesis or homeostasis of G_olf_ in the cell body.

We probed the cilia and OE immunoblots with additional antibodies, trying to detect links between the mislocalization of G_olf_ caused by the LRBA-KO, and known molecules and mechanisms involved in G_olf_ trafficking or in the ciliary targeting of other proteins (Fig. 4E–H). The adaptor protein CEP290/NPHP6 is implicated in the ciliary targeting of proteins including G_olf_ (refs 26, 27). CEP290 was detectable with similar signal intensities in cilia, OE and brain but unaffected by the LRBA-KO. We noted on this occasion that CEP290 proteins from OE and brain differ in size by ~50 kDa (Fig. 4E), suggestive of cell-type specific alternative splicing. Ric8B, an established interactor of Gα_olf_ and Gγ_13_ (refs 25, 27), also gave signals with cilia, OE and brain samples which, however, were unaffected by the LRBA-KO in cilia and OE. The integral membrane protein Stoml3/SLP3/SRO is implicated in mechanosensation and olfaction, its somato-dendritic distribution in OSNs^29^ is similar to LRBA, and its abundance is affected by KO of the Bardet-Biedl Syndrome proteins BBS1 and BBS4^30^. We found Stoml3 to be strikingly enriched in cilia compared to OE and brain, suggesting a physiological role in olfactory cilia, but independent of LRBA.

Gγ_13_ and the LRBA paralog, NBEA, have been found to bind to various PDZ domain scaffolding proteins: PSD95, SAP97, Veli-2, MPDZ/MUPP1, ZO-1, PIST/GOPC^31, 32^, and SAP102^33^, respectively, and we therefore probed for several proteins containing PDZ domains. PIST and Nherf1, as well as the Nherf1 interactor radixin, gave similar signal intensities with cilia, OE and brain samples, but were unaffected by the LRBA-KO. PSD95, SAP102 and MAGI-2/S-SCAM gave bands of appropriate sizes in the brain lanes, but were undetectable in OE and cilia.

IFT20 is implicated in the transport of cilia-destined membrane proteins from the Golgi complex to the cilium base. However, IFT20 signals, of similar strength in the cilia and OE lanes, were unaffected by the LRBA KO. Retinitis pigmentosa-2 (RP2) has an N-terminal dual acylation motif (MGCFFS…) similar to those of Gα_olf_ and Gα_s_ (refs 34–36) and is involved in Golgi-to-cilium vesicle transport and Gβ_1_ trafficking^37^. However, RP2 abundance was unaffected by the LRBA KO in olfactory cilia or OE. We noted that the main RP2 band in brain migrated slightly slower than the single RP2 band in cilia and OE, whereas a minor second band in brain co-migrated with the single band in cilia/OE (Fig. 4E, bottom), suggestive of splice-variant or posttranslational modification heterogeneity of RP2.

### Recruitment of LRBA to Golgi complex-near endomembranes is stimulated by GTPγS and antagonized by brefeldin A

Many steps of protein traffic are under the control of small GTPases. In particular, “coat proteins” (AP-1, AP-3, AP-4, COPI, GGAs) involved in cargo sorting and vesicle budding from various endomembrane compartments are under the control of Arf1. Arf1, in turn, is regulated by guanine nucleotide exchange factors (GEFs), some of which can be inhibited by brefeldin-A (BFA). Robinson and Kreis^38^ and Seaman *et al*.^39^ developed an *in-vitro* assay in which the de-novo recruitment of cytosolic coat proteins can be demonstrated, in its dependence on GTP and potentially its sensitivity to BFA. In this assay, “host” cells are permeabilized, and their cytosol is washed out while their endomembrane structures remain behind. These cell ghosts are then incubated with exogenous cytosol (isolated e.g. from mouse brain), and cytosolic coat proteins, as well as proteins associating with them, will be driven by GTPγS to bind to their appropriate target membranes, where they can be detected by IF or immunoblotting. Proteins recruited de-novo from the exogenous cytosol stand out particularly if the antibodies used for their detection are species-selective and recognize, as in our case, only the exogenous cytosolic protein but not the orthologous host cell protein (here, monkey COS7 cells).

**Figure 5:**
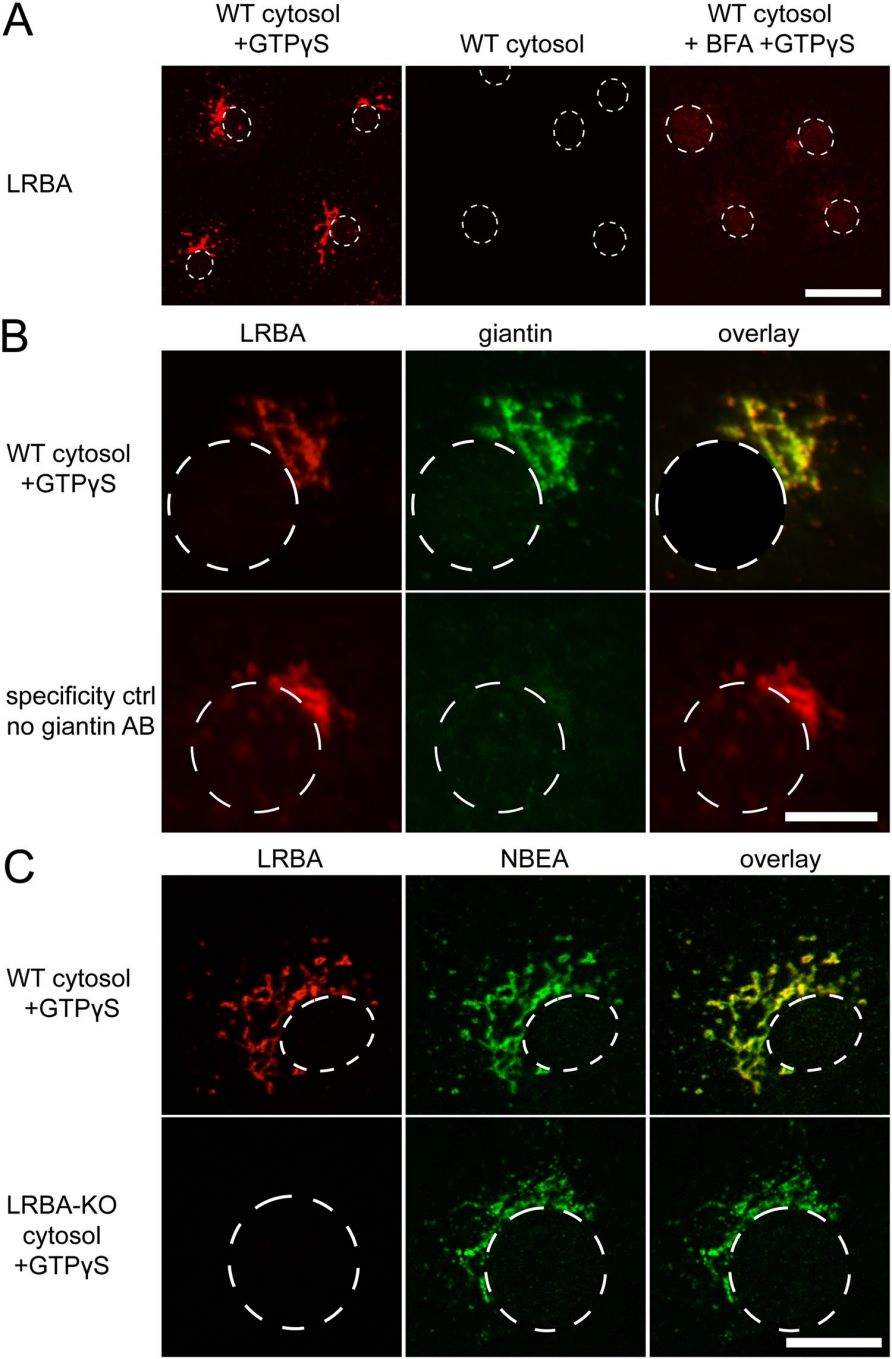
*In-vitro* recruitment of LRBA from brain cytosol to Golgi-near endomembranes of COS7 cell ghosts, observed by IF. (**A**) Recruitment of LRBA is stimulated by GTPγS and antagonized by BFA. (**B**) GTPγS-recruited LRBA co-localizes with giantin. Both primary antibodies were from rabbit; the giantin antibody was applied after the LRBA primary and secondary antibody, and subsequent blocking with anti-rabbit F_ab_ fragments. The control without giantin antibody confirms the blocking efficiency. (**C**) GTPγS-recruited LRBA and NBEA co-localize. Using cytosol from LRBA-KO brain, the LRBA signal is selectively lost. Scale bars: (**A**) 30 µm; (**B**,**C**) 15 µm. Cell nuclei, visible at higher exposures, are indicated by dashed outlines.

We previously demonstrated in this assay that NBEA is recruited from cytosol to Golgi complex-near endomembranes in a way reminiscent of coat proteins, stimulated by GTPγS and antagonized by BFA^3^. We now investigated whether LRBA exhibits the same behaviour. Indeed, we observed the recruitment of LRBA to perinuclear ribbon structures in the presence of GTPγS, but not in the absence of GTPγS (Fig. 5A), or when BFA was added prior to GTPγS (Fig. 5A), or in the presence of GDP (not shown). Double-IF demonstrated virtually perfect subcellular co-localization (at confocal IF resolution) of recruited LRBA with giantin, a marker for the Golgi membranes of the COS7 host cells (Fig. 5B), or with NBEA co-recruited from the cytosol (Fig. 5C). Using brain cytosol from LRBA-KO mice abolished the LRBA IF signal while leaving the NBEA signal unaffected (Fig. 5C), confirming the mutually exclusive specificities of the LRBA and NBEA antibodies in IF. This GTPγS-driven *in-vitro* recruitment of LRBA and NBEA probably labels the source compartment(s) of the putative transport pathways in which these two proteins participate, but it is not necessarily coincident with the predominant localizations of LRBA and/or NBEA at steady state in live cells. It suggests that GTP-dependent membrane recruitment may be a general property of BEACH proteins, and strengthens the notion that LRBA, similar to NBEA, is involved in membrane protein dynamics originating at or near the Golgi complex.

### No abnormalities detected in LRBA-KO mouse retina

The cell biologies of the sensory cilia of OSNs and of photoreceptor neurons are highly homologous, including the involvement of heterotrimeric G-proteins (G_olf_, transducin) in sensory signal transduction. Accordingly, ciliopathic gene defects in KO mice and humans can affect both olfaction and vision^30, 40, 41^. Rod α-transducin (Gαt1) is N-myristoylated at glycine-2, and its translocation from the rod/cone inner to the outer segment requires Unc119^35^, whereas γ-transducin (Gγ1) is C-terminally prenylated, and its rod/cone outer segment targeting involves the Unc119 homolog, PDE6D (PrBPδ)^42^, as solubilizing factors chaperoning the respective lipid anchors. Moreover, transducin and G_olf_ share the same β subunit isoform, Gβ1. We therefore explored whether LRBA-KO mice also display a retinal phenotype.

**Figure 6:**
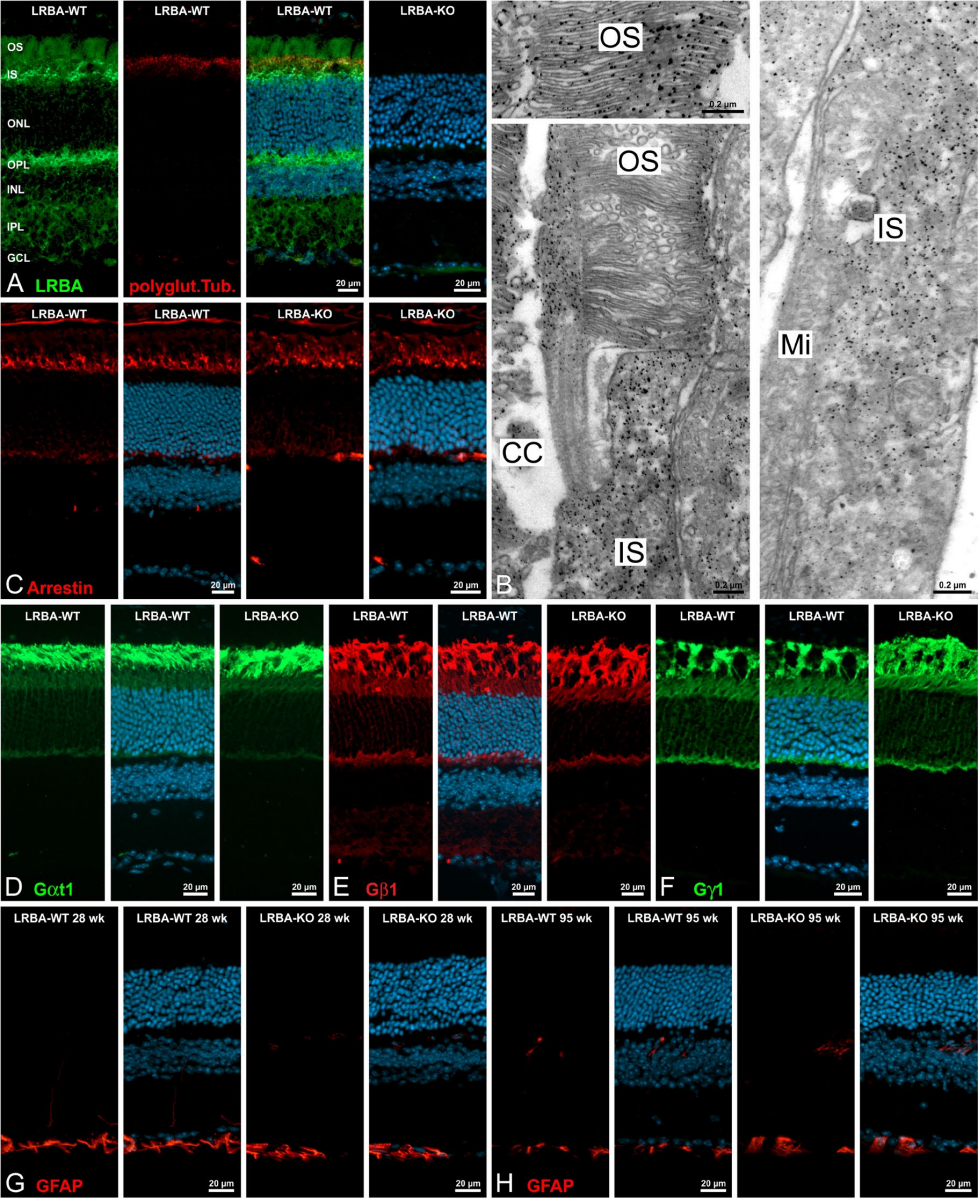
Localization of LRBA and histological analysis of the LRBA-KO in the mouse retina. (**A**) Micrographs of a vertical cryostat section through an adult WT mouse retina triple-labeled for LRBA (green), glutamylated tubulin (marker for connecting cilia, red) and DAPI (cell nuclei, blue). LRBA-ir is prominent in the outer segment (OS), the inner segment (IS) and the outer plexiform layers (OPL), and is abolished in KO mouse retina. ONL: outer nuclear layer; INL: inner nuclear layer; IPL: inner plexiform layer; GCL: ganglion cell layer. (**B**) Electron micrographs showing the ultrastructural localization of LRBA-ir (pre-embedding labeling) in the cytosol of outer and inner segments of rod photoreceptors. CC: connecting cilium; Mi: mitochondria. (**C**) Cryostat sections through light-adapted (2 h, 150–200 lux) WT and LRBA-KO retinae were stained with anti-arrestin (red) and DAPI (cell nuclei, blue). No differences were seen between the localizations of arrestin in the WT and LRBA-KO retinae. (**D–F**) Cryostat sections through light-adapted (2 h, 150–200 lux) WT and LRBA-KO retinae were stained with antibodies against the subunits α_t1_ (green), β_1_ (red) and γ_1_ (green) of transducin. Again, no differences were detected between the localizations in the WT and LRBA-KO retinae. (**G–H**) WT and LRBA-KO retinae of 28 and 95 week (wk) old mice were double-labeled for GFAP (green) and DAPI (blue). No up-regulation of GFAP in retinal Müller cells as an early indicator of retinal stress was detected.

Immunoblot analysis showed that the LRBA expression level in WT mouse retina is higher than in brain (Fig. 2J), and IF microscopy (Fig. 6A) detected marked LRBA staining of the inner and outer segment layers (IS, OS), and of the outer plexiform layer (OPL), whereas sparser staining was seen in the other layers of the retina. This LRBA-ir was abolished in KO mouse retina, except for a weak background signal in the ganglion cell layer (GCL; Fig. 6A right). Pre-embedding immuno-electron microscopy (EM) confirmed LRBA-ir throughout the cytoplasm of OS and IS of rod photoreceptor cells, sparing OS discs and IS mitochondria (Fig. 6B). The peripheral cytosol of OS was always decorated (large image) whereas the cytosol between discs was only occasionally labeled (small image), presumably due to poor labeling accessibility and /or small volume of the interdiscal spaces. However, no obvious association of LRBA-ir with subcellular structures of the IS such as endomembrane vesicles was noted. LRBA-KO mouse photoreceptor cells, processed in parallel, remained unlabeled in immuno-EM, and no ultrastructural anomalies were noted in EM (not shown). IF microscopy of retina, adapted to low light (200 Lux), for arrestin (Fig. 6C) and the three subunits of transducin (Fig. 6D–F) displayed the intermediate distributions of all four antigens between OS, IS, ONL and OPL, that are typical at this illumination intensity. No differences of labeling intensities or distributions were noted between WT and LRBA-KO mice.

Functional analysis by electroretinography (ERG) also failed to detect significant anomalies (Fig. S2A,B). Flash ERGs were analyzed in terms of amplitudes and implicit times of their key components (scotopic: a-wave, b-wave and oscillatory potentials [OPs]; photopic: b-wave, OPs), each reflecting the functional integrity of different retinal constituents (i.e. a-wave, photoreceptors and OFF-bipolar cells; b-wave, ON-bipolar cells; OPs, inner retina feedback involving amacrine and ganglion cells)^43^. Neither the scotopic nor the photopic flash ERG components from LRBA-KO mice differed from those recorded from WT mice (all *p* > 0.05). Flicker ERGs, which reflect other post-receptoral mechanisms^44^, were Fourier-analyzed to obtain amplitude and phase data of their fundamental components, and were also similar between the two groups. Only for signals modulated with 80% contrast at 12 Hz, the fundamental amplitude of LRBA-KO flicker ERG appeared smaller (strain * frequency, post-hoc *p* = 0.05). The phases of these and of all other flicker conditions were otherwise unchanged (all *p* > 0.05). The 12 Hz flicker ERG, if indeed significant, would reflect an impairment in ‘sustained’ post-receptoral mechanisms if the mouse ERG to sinusoidal modulation reflects similar post-receptoral processes as in the primate and human, analogous e.g. to the parvocellar pathway^44^.

Deficiencies of photoreceptor proteins may lead to retinal degeneration only in older mice, e.g. of PDE6D^42^, Cacna1f^45^, Unc119, usherin, or protein 4.1 G. However, neither in 6 months old nor in 22 months old mice could we observe effects of the LRBA-KO on the retinal layer architecture or on GFAP up-regulation in Müller glia cells (Fig. 6G,H), the latter known as an early sign of retinal degeneration^42, 45^. In conclusion, rod photoreceptor cells and other cell types of the retina do express LRBA, but unlike OSNs they can apparently tolerate or compensate the loss of LRBA, without an acute or chronic phenotype that would be recognizable by the molecular, electrophysiological and histological criteria addressed here.

## Discussion

Despite the marked cellular or organismic deficiency phenotypes of several BEACH proteins, an understanding of the molecular mechanistic pathways through which these proteins function is still lacking. The cumulated evidence points to roles in membrane dynamics and the subcellular targeting of multiple membrane proteins^1^. Findings with cells of human LRBA-deficient patients suggest that lack of LRBA perturbs the balance between plasma membrane recycling versus lysosomal degradation of the immune receptor CTLA-4 at a Rab11-positive endosomal compartment^11^, reminiscent of previous observations on LYST^46, 47^. However, involvements of plant BEACH proteins in the targeting of luminal proteins of the storage vacuole^48^, and of a protein component of cytosolic ribonucleoprotein particles^49^, suggest that the roles of BEACH proteins in subcellular protein traffic may be even more diverse and complex, extending also to non-transmembrane proteins.

Here, we report a physiological and molecular phenotype in LRBA-KO mice outside the immune system. We detect abnormalities of olfactory chemoreception at cellular and behavioural levels and show that LRBA is highly expressed in olfactory and vomeronasal sensory neurons. Finally we demonstrate, by two different techniques, markedly reduced levels of all three subunits of the peripheral membrane protein G_olf_ in the sensory cilia of OSNs, whereas the ciliary levels of other G-protein subunit isoforms, of several other ciliary proteins of the olfactory signal cascade and of some of their known or potential interactors were not or only marginally affected. The olfactory LRBA-KO phenotype appears more pronounced at the molecular than at the cellular and behavioural levels. Whereas G_olf_ subunit abundances in cilia of adult mice were reduced by 60-80% in immunoblot analysis, the odorant-induced EOG response decreased only by 16-30%, and also the olfaction-driven behavioural phenotype was mild. This suggests that normal G_olf_ levels are not immediately limiting for signal strength and that, although in Gα_olf_ *null* mice the EOG amplitude is drastically reduced (down to <2.5% in adults) and EOG response is delayed^50^, the residual 20-40% levels of G_olf_ in the LRBA-KO mice are still sufficient to maintain 70-84% EOG amplitude and normal response kinetics. We did not observe a compensatory up-regulation of other proteins of the olfactory signal chain in cilia (Fig. 4), or of the LRBA paralog NBEA in the brain of LRBA-KO mice (Fig. 1B).

The KO effect of Gα_olf_ itself on EOG amplitude was reported to be weaker in neonatal than in adult mice (25% vs. 2.5% residual response^50^), possibly due to larger contributions of G_s_ (refs 51, 52) and G_o_ (ref. 53) to olfactory signalling in younger OSNs. In contrast, the impact of the LRBA-KO on EOG seems to be stronger in e18 than in adult mice (Fig. 2B,C). This may be due to an involvement of LRBA in the functional expression also of G_s_ (as the reduced Gα_s_-S signals in LRBA-KO cilia would suggest [Fig. 4E,F]), or to better compensation of LRBA deficiency in the adult olfactory system. It is unknown whether BEACH proteins have any functional overlap. We detected no significant additional reduction of the EOG response of e18 LRBA/NBEA-DKO mice (Fig. 2B), but NBEA might become more critical for mature OSNs. It is therefore possible that the residual G_olf_ level or the largely unaffected levels of other olfactory signal proteins, which we determined in the adult LRBA-KO mice, would be reduced further in olfactory cilia of an adult, conditional LRBA/NBEA-DKO. As our constitutive NBEA-KO line is neonatal-lethal, we could not investigate this.

We detected prominent LRBA expression in both the apical and basal layers of vomeronasal sensory neurons (Fig. 3B) consistent with LacZ staining of both the anterior and posterior parts of the accessory olfactory bulb (Fig. 3C) to which these neuron populations respectively project. This encourages future investigations into vomeronasal LRBA-KO phenotypes at the functional and molecular levels. Our observation that male aggression is mildly reduced in LRBA-KO mice, even though the C57Bl/6N background is relatively insensitive to genetic traits affecting aggression in the resident-intruder paradigm^54^, also encourages further analysis of their vomeronasal function. However, mouse social behaviour can also be influenced by olfactory impairment alone^55–57^.

The clinical phenotype of human LRBA-mutant patients is dominated by severe immune deficiency^10–13^, but we and the German Mouse Clinic did not detect immunological symptoms in our LRBA-KO mice under pathogen-free maintenance conditions. Species differences of immune functions are not uncommon^47, 58^. Very recently, the immunological characterization of an independent LRBA-KO mouse line was published^14^. These mice carry a different mutation (gene-trap in intron 2) in a different genetic background (129 Sv) but, like ours, were viable and fertile without overt immunological symptoms. However, challenging their immune system by bone-marrow transplantation revealed compromised graft rejection, and impaired signalling of some immune receptors of Natural Killer cells. Hence, closer scrutiny of the immunological properties of LRBA-KO mice, particularly under challenge, can indeed uncover immunological abnormalities, contribute to understanding the mechanisms underlying the human LRBA-mutant immunodeficiency syndrome, and explore therapies.

According to Fig. 4, LRBA-KO mouse olfactory cilia have residual Gα_olf_, Gβ_1_ and Gγ_13_ levels of 20–40% by immunoblot analysis. Presumably, contamination of the purified cilia with other membranes from OE contributes to these residual levels. However, IF microscopy shows (Fig. 4A,B) that Gα_olf_ and Gβ_1_ of LRBA-KO mice still concentrate in the cilial layer, indicating that a lack of LRBA substantially reduces by not entirely abolishes the concentration of G_olf_ in olfactory cilia. The residual signals of Gα_olf_ and Gβ_1_ in the cilial layer determined by IF microphotometry are even higher than those determined by immunoblot analysis, but they, in turn, may be overstated by unspecific background binding of IF reagents to the epithelial surface with its olfactory cilia, sustentacular cell microvilli, and mucus. The finding that the residual Gα_olf_ signal is lower than the residual Gβ_1_ (in both IF and immunoblotting) and Gγ_13_ signals may mean that the latter subunits are subject to higher levels of background, or it may indicate that Gα_olf_ is the primary target of LRBA deficiency whereas the β_1_ and γ_13_ subunits are affected indirectly as fellow travellers of the α_olf_ subunit to the cilium. Existing evidence mostly indicates that the γ subunits are limiting for the targeting and/or stability of heterotrimeric G-proteins^27, 59–62^. By analogy, Gγ_13_ seems more likely to be the G_olf_ subunit primarily affected by LRBA deficiency. However, Unc119 binding to the acylated α subunits of mouse rod transducin and *C*. *elegans* chemosensory G-proteins also affects the subcellular distributions of these proteins^35^, so that both Gα_olf_ and Gγ_13_ remain candidates for being the primary targets of the mechanistic pathway connecting G_olf_ to LRBA.

G_s_ seems to be the predominant G protein isoform in neonatal olfactory cilia, but is replaced by G_olf_ in adult mice^51, 52^. Our immunoblotting data (Fig. 4E,F) suggest that it is the short splice variant, Gα_s_-S, which is subject to LRBA-dependent ciliary localization and possibly involved in neonatal ciliary function. In our brain samples, Gα_s_-S constituted 15-20% of total Gα_s_, whereas in both OE and cilia samples it constituted 55-60% of total Gα_s_, and only the apparent ciliary abundance of Gα_s_-S was reduced by LRBA deficiency. Gα_s_-S, lacking one exon, is the closest structural relative of Gα_olf_, and it is plausible that it might be subject to similar subcellular targeting mechanisms. However, it remains possible that the LRBA-sensitive Gα_s_-S signal component in the cilia samples is an analytical artifact due to a residual cross-reactivity of the Gα_s_ antibody (see Methods) with Gα_olf_, which co-migrates with Gα_s_-S and is far more abundant in cilia.

Subcellular trafficking of heterotrimeric G-proteins, which associate with membranes through post-translationally added lipid anchors, involves the chaperoning of the newly synthesized subunits by cytosolic proteins such as Ric8, CCT, PhLP1 and DRiP78; lipidation and heterotrimer assembly on the ER and the Golgi apparatus; and vesicular transport to the plasma membrane (review: ref. 63). Interactions with their receptors and effectors^64–66^ or other transmembrane proteins such as LRP6^67^, and with lipid-binding proteins mediating their shuttling between different membrane compartments *via* the cytosol by chaperoning the lipid anchors^35, 42, 68^ contribute to their subcellular dynamics. LRBA may be involved at any of these stages of G_olf_ biosynthesis or ciliary targeting: (a) synthesis, lipidation and heterotrimer assembly of its subunits; (b) transport to the apical, periciliary plasma membrane in the dendritic knobs, via vesicle carriers or through lipid-anchor chaperoning by solubilizing factors such as UNC119a, UNC119b or PDE6δ/PrBPδ which act on heterotrimeric G-proteins as well as other lipid-anchored proteins^35, 36, 42, 69^; (c) sorting at the periciliary plasma membrane or the cilium base and dispatching for uptake into the cilium; (d) intra-ciliary transport (IFT); (e) retention in cilia. The subcellular localization of LRBA seen by IF of OSNs (apical cytoplasm of somata, dendrites, and dendritic knobs, but not cilia; Fig. 3D–K), in combination with the GTP-stimulated and brefeldin-A-sensitive recruitment of LRBA to Golgi-near endomembranes (Fig. 5), primarily suggests stage (b), an involvement in trafficking events between the Golgi apparatus and the apical plasma membrane and/or the cilium base. Probing our immunoblots for several proteins (CEP290, Ric8B, Stoml3, PIST, IFT20, RP2) implicated in Golgi-to-cilium transport of G_olf_ subunits or other lipidated proteins, however, did not detect any impact of the LRBA-KO on their abundances in olfactory cilia or OE comparable to that on the G_olf_ subunits, which would have implicated them in the mechanistic pathway linking LRBA and G_olf_ localization. We probed for many additional proteins of interest (including UNC119a, UNC119b, PDE6δ/PrBPδ, LRP6, INPP5E, NPHP3, Arl3, Arl13b, Axin, Polycystin-2, PACS1), but available antibodies were not specific or sensitive enough to detect them in our experimental setting.

Previous studies of the impacts of BEACH protein KOs mostly detected the mislocalization of transmembrane proteins, such as growth factor receptors and an immune receptor in the case of LRBA/Rugose^7, 9, 11^ and ionotropic neurotransmitter receptors in the case of NBEA^70^. Unexpectedly, the present study identified a lipid-anchored heterotrimeric G-protein, G_olf_, as a cargo protein whose proper subcellular localization requires LRBA, thus adding to the pleiotropy of BEACH proteins. A few other proteins of olfactory cilia seem weakly reduced in abundance by the LRBA-KO (including another non-transmembrane protein, PDE1C, which we see reduced by IF as well as immunoblotting) (Fig. 4). These may be additional proteins genuinely dependent on LRBA, or their reduced ciliary abundance may be secondary to the effect on G_olf_. Given the expression of LRBA in many tissues and cell types not known to express G_olf_, more proteins probably require LRBA. It remains to be clarified by which mechanisms BEACH proteins influence the subcellular localizations of their known cargo proteins, and whether their effects on transmembrane proteins are sequential or parallel to those on G-protein(s) and perhaps other lipid-anchored proteins.

The molecular phenotypes of BEACH protein KOs are typically complex (i.e. affecting the localizations of multiple proteins^46, 70^) but partial (i.e. reducing but not abolishing correct localization; e.g. refs 16, 70, and the present study). This suggests that they are not part of the core machinery of protein sorting or targeting, but rather act to regulate or sharpen the functioning of this machinery. Identifying the molecular function of the BEACH domain itself will be pivotal for understanding the mechanistic principle of this protein family, which appears to constitute a novel dimension in the orchestration of subcellular protein traffic.

## Methods

### Gene-modified mice

Mice were generated, maintained and analyzed under approval of the Swedish and German animal welfare authorities, and in accordance with the respective animal welfare regulations. LRBA-KO mice (official allele designation, *Lrba* ^*tm1*.*1Kili*^ MGI:5796558; laboratory designation, *Lrba2*) were constructed by TaconicArtemis (Köln, Germany), through homologous recombination and screening of ES cell clones by Southern blot hybridization with four restriction enzymes and three probes. ES cell line A-C9 was injected into blastocysts to generate a conditional mouse line, whose mating with Rosa26(SA-Cre pA) deleter mice then generated a constitutive deletion of coding exon 3 (ATCTT…AATGG) (Fig. 1C). The Cre transgene was subsequently bred out. The first and last 5 nucleotides (nt) of exons and introns are specified here because exon numbering of the LRBA gene in the databases has been inconsistent. This deletion causes a frameshift after 149 of 2851 codons. The deletion was verified in *Lrba2* brain mRNA by RT-PCR between coding exons 1 and 5 with primers 5′-GTGCTGACGGGGTTGGTTGAA-3′ and 5′-GGATCCATTCTAAGCCAGGTGTGA-3′. For genotyping of mouse genomic DNA, PCR with primers 5′-GAAAGTTGACAGTATGATTGCAGG-3′ (forward, in coding exon 2) and 5′-CATTGTCCTTTATCTCCTTGAA-3′ (reverse, in coding exon 3) generates a WT product of 528 nt, whereas the same forward primer in combination with 5′-CTAAGGAGGATGGCTCTAACC-3′ (in intron 3) as reverse primer yields a product of 377 nt from the mutant allele (with coding exon 3 deleted). *Lrba2* mice were generated from an ES cell line of C57BL/6N origin, and had been backcrossed with C57BL/6N mice for five or more generations at the time of analysis.

Gene-trap mice expressing a LRBA-β-galactosidase fusion transcript under the control of the *Lrba* gene promoter were generated from ES cell line XE315 provided by BayGenomics (San Francisco), a member of the International Gene Trap Consortium (official allele designation, *Lrba* ^*Gt(XE315)Byg*^ MGI:4124772; laboratory designation, *Lrba/gt*). The gene trap vector pGT21xf had inserted into intron 38 (7693 nt long; gtcag…ttcag) of the *Lrba* gene. The interval around the vector was amplified from ES cell DNA and sequenced, localizing the integration site 750 nt downstream of exon 38 (125 nt long; TCGTC…ACATG) (Fig. 1D). The first 1210 nt of the gene trap vector had been lost during integration. The sequence loss was upstream of the splice acceptor site, but gene-trap efficiency was apparently compromised as the *Lrba/gt* mice retained residual expression levels of normal-sized LRBA of ~25% according to immunoblot analysis of brain and kidney. Blastocyst injections of the XE315 ES cells and implantation into foster mothers were carried out by the Uppsala University Transgenic Facility (UUTF), and the *Lrba/gt* allele was subsequently propagated in the C57BL/6N background. PCR genotyping with primers Lrba-ex38 F (5′-TAAGTCGTCCTCGTGAGTTC-3′) and Lrba-int38R (5′-AGGCCAAACCCAAGACTACA-3′) yields a WT product of 1131 nt, whereas the same forward primer combined with reverse primer Lrba-Trap5′R (5′-CTATACGAACGGTAGGATCC-3′) generates a mutant product of 920 nt. *Nbea* gene-trap KO mice (also in the C57BL/6N background) were described previously^16^ (official allele designation, *Nbea* ^Gt(RRK418)Byg^ MGI:4130191). Generation of *Lrba/Nbea*-DKO mice required extensive breeding effort, as both genes reside closely linked (~10 cMorgan) on mouse chromosome 3.

### EOG recordings

The skull was cut parasagitally to the septum in postnatal mice. In prenatal mice, part of the nose was cut off with a syringe needle tip to gain access to the nasal cavity. The turbinates were removed and EOGs were recorded from the olfactory epithelium on the septum. A constant humidified air stream was delivered to the olfactory epithelium (2.4 l/min). Odorant pulses of 100 ms were injected into the air stream via a custom-made device. Henkel 100 (Henkel, Düsseldorf, Germany), a mixture of 100 different odors^71^, was used as a stimulus. Rise times represent the timespan from 10 to 90% of the maximal amplitude, decay times from 90 to 10%.

### Animal behaviour tests

Before cookie-finding tests, male mice were housed in individual cages 1 week prior to the experiments. Mice had to find a cookie (Leibniz Butterkeks; Bahlsen, Hannover, Germany) buried beneath 6 cm of woodchip bedding in their home cage. The latency to locate the cookie was recorded. We defined the time to find the cookie as the time until the mouse held it in both fore-paws. If a mouse did not find the cookie after 15 min, the test was aborted and most bedding above the cookie was removed, so that the mouse could find it easily. The location of hiding was changed every day, and the cookie was never placed directly in a corner. Before the resident-intruder test of Fig. 2G, male mice >3 months of age were housed individually for 5 weeks. Before the test, the bedding of the resident mouse cage was not changed for 3 days. Intruder mice, also >3 months of age, were placed into the home cage of a resident mouse for 10 min. Within- and between-group comparisons of all truncated behaviour data were made using Mann-Whitney U test, other data with Student’s t-test.

### Antibodies

Mouse LRBA aa 942–1296 (KGD…RDS; encoded by coding exon 22 and the first 24 codons of exon 23; construct mLRBA-B) were cloned into the SmaI site of the His-tag vector pQE32 (Qiagen). Rabbit sera against the fusion protein were produced by Pineda Antibody Service (Berlin), and the sera were affinity-purified on the fusion protein used for immunization. Results of Figs 1, 2, 3, 4, 5 and 6B (immuno-EM; dilution 1:300) were obtained with anti-mLRBA-B antibody fractions purified from rabbit #1 (bleedings 38-39); IF results of Fig. 6 were generated with antibodies purified from rabbit #2 (bleeding 42; dilution 1:50-200), as the antibodies from rabbit #1 gave IF background labeling in retina specimens. As LRBA region B has low sequence conservation between species, our antibodies detect mouse but not human or monkey (COS7 cell) LRBA. Anti-NBEA antibodies against the collinear NBEA-specific sequence, region B, have been described^3^. Antibodies against TMEM16B^72^ and Olfr-73 (mOR-EG) were raised against specific peptide epitopes by Eurogentec (Seraing, Belgium). A rabbit serum against human giantin was a gift of M. Renz (Karlsruhe, Germany). The Gα_olf_-specific Ab (186/35) was made in the Herve´ laboratory^73^ by immunization against full-length recombinant Gα_olf_, affinity-purification on the same, and subtraction with full-length Gα_s_. Conversely, the Gα_s_ Ab (004/49) was made by immunization and affinity-purification with full-length Gα_s_ and subtraction with Gα_olf_.

The following other primary antibodies were employed successfully for immunoblot analysis. Mouse monoclonals: anti-Acetyl-tubulin (Santa Cruz sc-23950 1:100,000), anti-CNGA4/rOCN2 (Developmental Studies Hybridoma Bank DSHB-7B11 supernatant 1:50), anti-NCX1 (Swant [Marly/Switzerland] R3F1 1:33), anti-NKCC1 (DSHB-T4 supernatant 1:250), anti-pan-cadherin (Sigma C1821, clone CH-19 1:500), anti-PSD95 (NeuroMab K28/43 purified 1:12,500). Rabbit polyclonals: anti-Nherf1/EBP50 (Abcam ab3452 1:1000), anti-Stoml3 (Protein-Tech 13316 1:250), anti-Gβ_1_ (Santa Cruz sc-379 1:10,000), anti-Gβ_2_ (sc-380 1:100), anti-Gα_0_ (sc-387 1:200), anti-Gα_olf/s_ (sc-383 1:50,000), anti-Ric8B (Sigma 2106052 1:100, or Proteintech 17790 1:400), anti-CNGA2 (Sigma N7529 1:500), anti-CNGB1^74^ (1:10,000), anti-PDE1C (sc-67323 1:2000), anti-TMEM16B^72^ (1:2000), anti-ACIII (sc-588 1:2000), anti-CEP290 (Novus 86991 1:1000), anti-SAP102 (Synaptic Systems 124202 1:1000), anti-MAGI-2/S-SCAM (Sigma M2441 1:250), anti Gγ_13_ (Aviva ARP56936 1:1000), anti-Radixin (Sigma R3653 1:500), anti-PIST (Sigma SAB3500332 1:600), anti-RP2 (Proteintech 14151 1:500), anti-IFT20 (Proteintech 13615 1:400). Goat polyclonal: anti-OMP (Wako 544–10001 1:100,000).

We only evaluated immunoblot bands whose migration was consistent with the predicted MWs and/or the literature, to ensure specificity. Proteintech anti-Ric8B (17790) labeled two bands on OE and cilia immunoblots (~60 and 70 kDa; unlike Sigma 2106052 which labels only the 60 kDa band) and an additional 65 kDa band in brain (Fig. 4E,G). Developing an immunoblot of OE from WT and Ric8B-KO mice (courteousy of B. Malnic) confirmed that both bands are abolished in Ric8B-KO OE and therefore represent Ric8B.

### IF microscopy and LacZ histochemistry of olfactory tissues

Mice were deeply anesthetized and killed by cervical dislocation or decapitation. For IF on cryostat sections of OE (Fig. 4A–C), heads were fixed in 4% paraformaldehyde (PFA) in phosphate-buffered saline (PBS) overnight at 4 °C, then placed in 30% sucrose/PBS for 12 hours. Cryosections (14μm) of mouse OE were prepared and mounted on Superfrost glass slides (Menzel Gläser, Braunschweig, Germany). Sections were permeabilized and blocked in IF blocking buffer (1% cold-water fish skin gelatin, 0.1% Triton X-100 in PBS) prior to incubation with primary antibodies diluted in IF blocking buffer. Primary antibodies were applied overnight at 4 °C, secondary antibodies for 30 min at RT. The following primary antibodies were employed successfully (all from Santa Cruz): Gα_s/olf_ sc-383 (1:300), CNGA2 sc-13700 (1:50), ACIII sc-588 (1:300), acetylated tubulin sc-23950 (1:50), PDE1C sc-67323 (1:100), Gβ_1_ sc-379 (1:50). As secondary antibodies, goat anti-rabbit conjugated to Alexa 568 (Molecular Probes A11010), donkey anti-goat conjugated to Alexa 633 (Molecular Probes A21082), and goat anti-rabbit conjugated to Alexa 633 (Molecular Probes A21071) were used (1:1000). Sections were mounted in ProLong Gold Antifade (Molecular Probes). All IF images of ciliary layer cross-sections are single plane XY images and were obtained with a Leica DM6000 confocal microscope setup. For quantification, two cryosections each from 3 WT and 3 KO mice of mixed sex (age, 14d) were double-stained for acetyl-tubulin and each protein of interest (specificity controls: omission of primary antibodies). From each section, two 60 µm^2^ frames were selected from dorsal OE areas lining the septum, taken with the same settings, and fluorescence intensities measured and analyzed with the Leica Application Suite 2.1.1.

For whole-mount immunostaining of cilia (Fig. 4D), turbinates were fixed in 4% PFA/PBS for 30 min, blocked with IF blocking buffer for 30 min, and processed for IF with mOR-EG primary antibody (1:100) as described above. Z-Scans of individual areas were performed, merged, and analyzed with the Leica Application Suite 2.1.1.

Triple-IF labeling (Fig. 3D–K) was carried out on semithin sections from the OE of adult WT mice. The nasal mucosa was carefully dissected^75^, immediately frozen in melting isopentane cooled with liquid nitrogen, freeze dried and embedded in Epon^76^. Semithin sections (~1 µm) were cut from the embedded material, mounted on glass coverslips, etched^76^ and transferred to PBS. Sections were then covered with 50-100 µl of the appropriate dilution of the primary antisera or their combinations in incubation buffer (PBS containing 0.5% normal donkey serum [Sigma] and 0.05% sodium azide) and incubated overnight at 4 °C in a humid chamber. Antibodies used were rabbit anti-mLRBA-B (1:100), goat anti-OMP (1:300; Wako) as a marker for mature OSNs^22^, and mouse anti-GAP43 (1:300; MerckMillipore) as a marker for immature OSNs^22, 77^. After washing in PBS, the sections were incubated in the appropriate secondary antisera [donkey anti-mouse-Cy3, donkey anti-rabbit-Cy2, donkey anti-goat-7-Amino-4-methylcoumarin-3-acetic acid (AMCA); all Dianova, Hamburg, Germany] diluted 1:200 or 1:600, or combinations of these, in incubation buffer for 2–4 h. Sections were again washed, dehydrated in graded ethanol, mounted in DePex (Serva), and observed with a Zeiss Axioskop fluorescence microscope. Each reaction was done repeatedly, and controls were carried out by omitting from the reaction mixture the primary antibody in single labelings, or either of the primary antibodies in triple labelings. No labeling over background or cross-reactivity of the reaction series was observed in controls.

For LacZ staining, cryosections of tissues from *Lrba/gt* and WT mice, aged 3 weeks, were incubated simultaneously in staining solution at 37 °C until staining was prominent. To prepare fresh staining solution, a stock solution of 40 mg/ml X-gal in DMSO was diluted 1:30 in 5 mM K_3_(Fe(CN)_6_), 5 mM K_4_(Fe(CN)_6_), 2 mM MgCl_2._

### Cilia purification

Each cilia preparation was derived from pooled OEs of 3–4 mice of mixed sex, aged 4 weeks. All solutions contained protease inhibitor cOmplete Mini (Roche, Mannheim, Germany). OE was washed in deciliation buffer (140 mM NaCl, 2 mM MgSO_4_, 7.5 mM D-glucose, 20 mM HEPES, 5 mM EGTA, 20 mM CaCl_2_, 30 mM KCl, pH 7.4) lacking KCl and CaCl_2_, and then suspended in 500 µl complete deciliation buffer. After 20 min incubation at 4 °C on a shaker at 300 rpm, the suspension was centrifuged at 6,600 rcf at 4 °C for 5 min. The supernatant containing the detached cilia was set aside, and the OE pellet was again resuspended in 300 µl deciliation buffer. This procedure was repeated four times. The five supernatants were pooled and centrifuged at 45,000 rcf, 4 °C, for 30 min to sediment the cilia. Cilia were finally resuspended in TEM buffer (10 mM Tris, 3 mM MgCl_2_, 2 mM EGTA, pH 7.4) and stored at −70 °C.

### Immunoblot analysis

For Fig. 1A,B, proteins were resolved on home-made 6% SDS-PAGE gels. LRBA and NBEA often failed to enter the gel efficiently if samples were prepared in conventional SDS/mercaptoethanol loading buffer and boiled. If blots were to be probed for LRBA or NBEA, we therefore used the following procedure derived from sample preparation for isoelectric focusing: mix 1 vol of tissue lysate with 3 vol of lysis buffer (6 M urea, 375 mM Tris pH 8.8, 2% SDS, 20% glycerol, 2.5% iodoacetamide), incubate at 30 °C for 30 min, then sonicate 30 min in an ultrasound bath; add bromophenol blue and apply to gel.

Cilia samples (Fig. 4E) were resolved on 4–15% Mini-PROTEAN TGX gradient gels (BioRad) and transferred to PVDF membranes (Millipore Immobilon-P) by semidry blotting. Equal protein quantities (~10 µg) of three distinct cilia preparations each from WT and KO mice were loaded in parallel on the same blot. A dilution series of a WT cilia preparation was included on each gel/blot to provide an internal calibration curve, to account for the limited linearity of the detection techniques (typically, 1.5fold to 0.25fold sample size to encompass the whole signal intensity range in the samples to be quantified; not shown in Fig. 4E). Samples of WT OE and/or brain homogenates (~50 µg) were also included on the cilia blots for reference. Immunodetection was performed either by chemiluminescence (ECL) or infrared fluorescence (LI-COR). For a few antigens (including Gα_s/olf_ and Gβ_1_), both detection techniques were used with concordant results. For ECL, membranes were blocked in WB blocking buffer (5% non-fat dry milk, 20 mM Tris pH 7.5, 150 mM NaCl, 0.1% Tween-20) at 4 °C overnight, and incubated with primary and secondary antibodies, each in WB blocking buffer at room temperature for 90 min. Chemiluminescence development was performed with the ECL-Plus or ECL-Prime kits (GE Healthcare). Chemiluminescence signals were collected on GE Hyperfilm-ECL films, scanned, quantified using the Quantity One software (Bio-Rad), and finally normalized on the acetyltubulin signals in the respective lanes to account for variability of loading or cilia purity. For infrared fluorescence detection, the following protocol was applied: block 60-120 min at room temperature in LI-COR blocking buffer; add 0.1% Tween-20 and the primary antibody, incubate at 4 °C overnight; wash 4 × 5 min in Tris-buffered saline (TBS)/0.1% Tween-20; incubate 60–120 min at 4 °C with the secondary antibody in 50% blocking buffer, 50% TBS, 0.1% Tween-20, 0.05% SDS; wash again, scan and quantify with the LI-COR Odyssey 9201 instrument equipped with Image Studio 3.1 software. For the electrophoretic resolution of some proteins, details of the sample preparation technique were critical. If samples were prepared in conventional SDS/mercaptoethanol loading buffer and boiled, CNGA2, CNGA4, CNGB1, ACIII and NKCC1 yielded heterogeneous band patterns difficult or impossible to quantify. When we followed the recommendation in ref. 78 to denature multi-pass transmembrane proteins at 55 °C for 1 h (in the presence of 5% mercaptoethanol and 100 mM DTT) rather than by boiling, these antigens indeed produced single bands of the expected molecular sizes and of much higher intensity.

For quantitative immunoblot analysis of OE (Fig. 4G), OE from 3 WT and 3 LRBA-KO mice of mixed sex (~5 mg wet weight from each mouse, aged 28d) was collected on dry-ice, powdered on liquid nitrogen, dissolved in SDS sample buffer, denatured 5 min at 95 °C, and then electrophoresed, blotted, developed with the LI-COR technique and quantified as described above.

### Recruitment of LRBA and NBEA to COS7 cell membranes

Experiments were carried out according to ref. 3, and IF staining performed as described above for cryosections. NBEA, LRBA and giantin primary antibodies were all from rabbit. For double IF, residual binding sites of the first primary antibody for secondary antibodies were blocked with goat anti-rabbit IgG, F(ab’)_2_ fragments (Santa Cruz, sc-3836) for 1 h at RT, before applying the next primary and secondary antibodies. In the control of Fig. 5B, a parallel mock-incubation without anti-giantin was followed by regular application of the green secondary antibody; the virtual absence of green labelling confirmed the blocking efficiency.

### Retina IF

Mice were maintained on a 12/12 h light/dark cycle (light on at 6 a.m.) and an average illumination of 150–200 lux (white light; TLD 58 W/25 tubes, Philips). Mice were light-adapted for 2 h after 12 h in the dark. The eyes were opened and retinae were immersion fixed in the eyecup for 15–30 min in 4% PFA in PBS. Eyecups were PBS-washed 3 times followed by increasing sucrose steps for cryo-protection. Eyecups were mounted in a cup filled with freezing medium (Sakura – Tissue-Tek, Staufen, Germany) and frozen immediately in pure isopentane cooled in liquid nitrogen for 45 seconds. Afterwards the frozen cups with the eyes were transferred directly into liquid nitrogen for a few minutes. The frozen cups can be stored at −80 °C until sectioning. The eyes were sectioned vertically with a cryostat (Leica CM3050S) at a thickness of 10–14 µm. The sections were collected on Superfrost Plus slides (Menzel Gläser, Braunschweig, Germany) for the following immunocytochemical stainings. The retinal sections were blocked for 1 h in blocking solution (10% normal goat serum (NGS), 1% bovine serum albumin (BSA), 0.5% Triton X-100 in PBS) and incubated in the primary antibodies (diluted in 3% NGS, 1% BSA, 0.5% Triton X-100 in PBS) overnight at RT. Rabbit polyclonal antibodies against Gα_t1_ (K-20; 1:500), Gβ_1_ (P-19; 1:1000) and Gγ_1_ (T-20; 1:1000) were all from Santa-Cruz. Monoclonal mouse antibodies against polyglutamylated tubulin (GT335; 1:1000) and rod arrestin (3D1.2; 1:100) were from Enzo Life Science, Lörrach, Germany. Next day, the samples were washed 3 × 10 min in PBS and incubated 2 h with secondary antibodies Alexa^TM^ 568 (red fluorescence) and Alexa^TM^ 488 (green fluorescence) goat anti-mouse, goat anti-rabbit IgG (H + L) conjugates (1:500; Molecular Probes) in antibody solution with DAPI (4,6-diamidino-2-phenylindole) (1:50000, Sigma-Aldrich). After further 3 × 10 min PBS washing steps, samples were mounted in Aqua Poly Mount (Polysciences, Eppelheim, Germany) and analyzed with a Zeiss Axio Imager Z2 equipped with a Zeiss ApoTome. Images were taken with a 20× (0.8, Apochromat) objective as stacks of multiple optical sections, and projections were calculated with the ZEN blue 2012 software (Zeiss). Images were adjusted for contrast and brightness using Adobe Photoshop CS6 (Adobe Systems, San Jose, CA, USA) and figures were arranged using Corel Draw X8.

### Retina pre-embedding immuno-EM

Subretinally injected retinae were prepared and stained as described previously^79^. Briefly, the dissected retinae were prefixed in 4% PFA for 50 min at RT. After four cycles of freezing and thawing, retinae were washed in PBS and embedded in buffered 4% Sieve 3:1 Agarose (Biozym). Agar blocks were sectioned at a thickness of ~100 μm with a Vibratome (Leica VT 1000 S). Vibratome sections were blocked in 10% NGS and 1% BSA in PBS for 2 h at RT and then incubated with primary antibodies for 4 days at 4 °C. After washing the retina 4x in PBS, the sections were subjected to corresponding biotinylated secondary antibodies and a peroxidase-based enzymatic detection system (Vectastain Elite ABC kit, Vector Laboratories). For immunocomplex visualization, 0.01% hydrogen peroxide (Sigma-Aldrich) was added to a 0.05% 3.3′-diaminobenzidine solution and staining was fixed in 2.5% glutaraldehyde (Sigma-Aldrich) in cacodylate buffer (0.1 M, pH 7.4) for 1 h at RT. Following silver intensification and dehydration, sections were flat-mounted in Epoxy Embedding Medium (Sigma-Aldrich). After sectioning on an ultramicrotome (Reichert Ultracut E), retina ultrathin sections (60–70 nm) were collected on Pioloform-coated copper grids and stained with uranyl acetate and lead citrate (Leica). Ultrathin sections were examined and photographed with a Zeiss EM10 electron microscope and a GATAN SC1000 OriusTM CCD camera with DigitalMicrograph^TM^ software (GATAN, Pleasanton, CA, USA). Representative images were chosen based on the inspection of several hundred slices from independent experiments. Micrographs were adjusted for contrast and brightness by using Adobe Photoshop CS6.

### Electroretinography (ERG)

Details of ERG recording in mice have been described previously^45, 80, 81^. In short, overnight dark-adapted mice (WT, n = 2; LRBA-KO littermates, n = 4; all female, mean age 5.3 ± 0.9 mo) were anesthetized (i.m. ketamine:xylazine, 50:10 mg/kg) and had their pupils dilated (1 gtt. each of tropicamide and phenylephrine-hydrochloride) for ERG measurements in a dark room. Further handling of animals was done under dim red illumination. Recording electrodes included corneal contact lens electrodes (actives), SC needles medial to ears (inactives) and on base of tail (ground). Once in place, the mouse positioned head-first in a Ganzfeld bowl (Roland Consult Q450) was subjected to three light protocols – (i) a scotopic flash series (0.0002 to 6.3 cd s/m^2^) to assess rod pathway function, (ii) a photopic flash series (0.02 to 6.3 cd s/m^2^ on a 25 cd s/m^2^ background), and (iii) sine-wave flickers (mean luminance 60 cd s/m^2^) presented at temporal frequencies of 12, 18, 24, and 30 Hz, for modulation contrasts 40, 60, 80, and 100%. Only white light-emitting diodes were used (programmed by RetiPort). A recording session lasted ~45 min. Two-way repeated measures (RM)-ANOVA were adopted to evaluate datasets across a series of flash strengths, and across temporal frequencies. Bonferroni post-tests were conducted when a significant interaction arose to evaluate parameter differences among individual intensities or frequencies.

### Statistical analysis

Unless indicated otherwise (Figs 2F–G and S2), datasets were evaluated by Student’s t-test. All p-values are two-tailed, and the bar diagrams depict mean ± SEM. In all figures: *p < 0.05; **p < 0.005; ***p < 0.001 relative to WT.

## Electronic supplementary material

Supplementary Figures S1, S2

## References

[1] Cullinane AR, Schäffer AA, Huizing M (2013). The BEACH is hot: A LYST of emerging roles for BEACH-domain containing proteins in human disease. Traffic.

[2] Olszewski PK (2012). Neurobeachin, a regulator of synaptic protein targeting, is associated with body fat mass and feeding behavior in mice and body-mass index in humans. PLoS Genet..

[3] Wang X (2000). Neurobeachin: A protein kinase A-anchoring, beige/Chediak-Higashi protein homolog implicated in neuronal membrane traffic. J. Neurosci..

[4] Wang JW, Howson J, Haller E, Kerr WG (2001). Identification of a novel lipopolysaccharide-inducible gene with key features of both A kinase anchor proteins and chs1/beige proteins. J. Immunol..

[5] Tsang WH, Shek KF, Lee TY, Chow KL (2009). An evolutionarily conserved nested gene pair - Mab21 and Lrba/Nbea in metazoan. Genomics.

[6] Shamloula HK (2002). rugose (rg), a Drosophila A kinase anchor protein, is required for retinal pattern formation and interacts genetically with multiple signaling pathways. Genetics.

[7] De Souza N, Vallier LG, Fares H, Greenwald I (2007). SEL-2, the C. elegans neurobeachin/LRBA homolog, is a negative regulator of lin-12/Notch activity and affects endosomal traffic in polarized epithelial cells. Development.

[8] Volders K (2012). Drosophila rugose is a functional homolog of mammalian Neurobeachin and affects synaptic architecture, brain morphology, and associative learning. J. Neurosci..

[9] Wang JW (2004). Deregulated expression of LRBA facilitates cancer cell growth. Oncogene.

[10] Lopez-Herrera G (2012). Deleterious mutations in LRBA are associated with a syndrome of immune deficiency and autoimmunity. Am. J. Hum. Genet..

[11] Lo B (2015). Patients with LRBA deficiency show CTLA4 loss and immune dysregulation responsive to abatacept therapy. Science.

[12] Alkhairy OK (2016). Spectrum of phenotypes associated with mutations in LRBA. J. Clin. Immunol..

[13] Gamez-Diaz L (2016). The extended phenotype of LPS-responsive beige-like anchor protein (LRBA) deficiency. J. Allergy Clin. Immunol..

[14] Park MY (2016). LRBA is essential for allogenic responses in bone marrow transplantation. Sci. Rep..

[15] Andres SA, Brock GN, Wittliff JL (2013). Interrogating differences in expression of targeted gene sets to predict breast cancer outcome. BMC Cancer.

[16] Medrihan L (2009). Neurobeachin, a protein implicated in membrane protein traffic and autism, is required for the formation and functioning of central synapses. J. Physiol..

[17] Su Y (2004). Neurobeachin is essential for neuromuscular synaptic transmission. J. Neurosci..

[18] Michalakis S (2006). Loss of CNGB1 protein leads to olfactory dysfunction and subciliary cyclic nucleotide-gated channel trapping. J. Biol. Chem..

[19] Coppola DM (2012). Studies of olfactory system neural plasticity: The contribution of the unilateral naris occlusion technique. Neural Plasticity.

[20] Weiss J (2011). Loss-of-function mutations in sodium channel Na_v_1.7 cause anosmia. Nature.

[21] Ying G (2014). Centrin 2 is required for mouse olfactory ciliary trafficking and development of ependymal cilia planar polarity. J. Neurosci..

[22] Schwob JE (2002). Neural regeneration and the peripheral olfactory system. Anat. Rec..

[23] Cygnar KD, Zhao H (2009). Phosphodiesterase 1C is dispensable for rapid response termination of olfactory sensory neurons. Nat. Neurosci..

[24] Billig GM, Pal B, Fidzinski P, Jentsch TJ (2011). Ca^2+^-activated Cl^−^ currents are dispensable for olfaction. Nat. Neurosci..

[25] Kerr DS, Von Dannecker LEC, Davalos M, Michaloski JS, Malnic B (2008). Ric-8B interacts with Gαolf and Gγ13 and co-localizes with Gαolf, Gβ1 and Gγ13 in the cilia of olfactory sensory neurons. Mol. Cell. Neurosci..

[26] McEwen DP (2007). Hypomorphic CEP290/NPHP6 mutations result in anosmia caused by the selective loss of G proteins in cilia of olfactory sensory neurons. Proc. Natl. Acad. Sci. USA.

[27] Li F (2013). Heterotrimeric G protein subunit Gγ13 is critical to olfaction. J. Neurosci..

[28] Herve D (1993). G_olf_ and G_s_ in rat basal ganglia: possible involvement of G_olf_ in the coupling of dopamine D_1_ receptor with adenylate cyclase. J. Neurosci..

[29] Goldstein BJ, Kulaga HM, Reed RR (2003). Cloning and characterization of SLP3: a novel member of the stomatin family expressed by olfactory receptor neurons. J. Assoc. Res. Otolaryngol..

[30] Kulaga HM (2004). Loss of BBS proteins causes anosmia in humans and defects in olfactory cilia structure and function in the mouse. Nat. Genet..

[31] Li Z, Benard O, Margolskee RF (2006). Gγ13 interacts with PDZ domain-containing proteins. J. Biol. Chem..

[32] Liu Z (2012). Identification of new binding partners of the chemosensory signaling protein Gγ13 expressed in taste and olfactory sensory cells. Front. Cell. Neurosci..

[33] Lauks J (2012). Synapse-associated protein 102 (SAP102) binds the C-terminal part of the scaffolding protein neurobeachin. PLoS One.

[34] Kleuss C, Krause E (2003). Gα_s_ is palmitoylated at the N-terminal glycine. EMBO J..

[35] Zhang H (2011). UNC119 is required for G protein trafficking in sensory neurons. Nat. Neurosci..

[36] Malicki J, Avidor-Reiss A (2014). From the cytoplasm into the cilium: bon voyage. Organogenesis.

[37] Schwarz N, Novoselova TV, Wait R, Hardcastle AJ, Cheetham ME (2012). The X-linked retinitis pigmentosa protein RP2 facilitates G protein traffic. Hum. Mol. Genet..

[38] Robinson MS, Kreis TE (1992). Recruitment of coat proteins onto Golgi membranes in intact and permeabilized cells: effects of brefeldin A and G protein activators. Cell.

[39] Seaman MNJ, Ball CL, Robinson MS (1993). Targeting and mistargeting of plasma membrane adaptors *in vitro*. J. Cell Biol..

[40] Nishimura DY (2004). Bbs2-null mice have neurosensory deficits, a defect in social dominance, and retinopathy associated with mislocalization of rhodopsin. Proc. Natl. Acad. Sci. USA.

[41] Jansen F (2016). Impact of the Usher syndrome on olfaction. Hum. Mol. Genet..

[42] Zhang H (2007). Deletion of PrBP/δ impedes transport of GRK1 and PDE6 catalytic subunits to photoreceptor outer segments. Proc. Natl. Acad. Sci. USA.

[43] Frishman, L. J. Origins of the electroretinogram. In: *Principles and Practice of Clinical Electrophysiology of* Vision (ed. Heckenlively, J. R. & Arden, G. B.) 139–183 (MIT Press, 2006).

[44] Kremers, J. Signal Pathways in the Electroretinogram. In: *Electroretinograms* (ed. Belušič, G.) 55–78 (Rijeka: InTech, 2011).

[45] Regus-Leidig H (2014). Photoreceptor degeneration in two mouse models for congenital stationary night blindness type 2. PloS One.

[46] Faigle W (1998). Deficient peptide loading and MHC class II endosomal sorting in a human genetic immunodeficiency disease: the Chediak-Higashi syndrome. J. Cell Biol..

[47] Barrat FJ (1999). Defective CTLA-4 cycling pathway in Chediak-Higashi syndrome: A possible mechanism for deregulation of T lymphocyte activation. Proc. Natl. Acad. Sci. USA.

[48] The OK (2015). BEACH-domain proteins act together in a cascade to mediate vacuolar protein trafficking and disease resistance in Arabidopsis. Mol. Plant.

[49] Steffens A, Bräutigam A, Jakoby M, Hülskamp M (2015). The BEACH domain protein SPIRRIG is essential for Arabidopsis salt stress tolerance and functions as a regulator of transcript stabilization and localization. PLoS Biology.

[50] Belluscio L, Gold GH, Nemes A, Axel R (1998). Mice deficient in G_olf_ are anosmic. Neuron.

[51] Chesler AT (2007). A G protein/cAMP signal cascade is required for axonal convergence into olfactory glomeruli. Proc. Natl. Acad. Sci. USA.

[52] Scholz P (2016). Transcriptome analysis of murine olfactory sensory neurons during development using single cell RNA-seq. Chem. Senses.

[53] Scholz P (2016). Identification of a novel Gnao-mediated alternative olfactory signaling pathway in murine OSNs. Front. Cell. Neurosci..

[54] Tantra M (2014). Mild expression differences of MECP2 influencing aggressive social behavior. EMBO Mol. Med..

[55] Mandiyan VS, Coats JK, Shah NM (2005). Deficits in sexual and aggressive behaviors in Cnga2 mutant mice. Nat. Neurosci..

[56] Glinka ME (2012). Olfactory deficits cause anxiety-like behaviors in mice. J. Neurosci..

[57] Matsuo T (2015). Genetic dissection of pheromone processing reveals main olfactory system-mediated social behaviors in mice. Proc. Natl. Acad. Sci. USA.

[58] Mestas J, Hughes CCW (2004). Of mice and not men: differences between mouse and human immunology. J. Immunol..

[59] Schwindinger WF (2003). Loss of G protein γ_7_ alters behavior and reduces striatal α_olf_ level and cAMP production. J. Biol. Chem..

[60] Lobanova ES (2008). Transducin γ-subunit sets expression levels of α- and β-subunits and is crucial for rod viability. J. Neurosci..

[61] Schwindinger WF (2010). Adenosine A_2A_ receptor signaling and G_olf_ assembly show a specific requirement for the γ_7_ subtype in the striatum. J. Biol. Chem..

[62] Calvert PD (2000). Phototransduction in transgenic mice after targeted deletion of the rod transducin α-subunit. Proc. Natl. Acad. Sci. USA.

[63] Wedegaertner, P. B. G protein trafficking. In *GPCR signalling complexes - Synthesis*, *assembly*, *trafficking and specificity*. (*ed*. Dupre’, D. J. *et al*.). 193–223 (Dordrecht: Springer Science + Business Media, 2012).

[64] Dupré DJ (2006). Seven transmembrane receptor core signaling complexes are assembled prior to plasma membrane trafficking. J. Biol. Chem..

[65] Robitaille M, Ramakrishnan N, Baragli A, Hébert TE (2009). Intracellular trafficking and assembly of specific Kir3 channel/G protein complexes. Cell Signal..

[66] Xie K (2015). Stable G protein-effector complexes in striatal neurons: mechanism of assembly and role in neurotransmitter signaling. eLife.

[67] Wan M (2011). LRP6 mediates cAMP generation by G protein-coupled receptors through regulating the membrane targeting of Gα_s_. Science Signal..

[68] Gopalakrishna KN (2011). Interaction of transducin with uncoordinated 119 protein (UNC119): implications for the model of transducin trafficking in rod photoreceptors. J. Biol. Chem..

[69] Chandra A (2012). The GDI-like solubilizing factor PDEδ sustains the spatial organization and signalling of Ras family proteins. Nat. Cell Biol..

[70] Nair R (2013). Neurobeachin regulates neurotransmitter receptor trafficking to synapses. J. Cell Biol..

[71] Wetzel CH (1999). Specificity and sensitivity of a human olfactory receptor functionally expressed in human embryonic kidney 293 cells and Xenopus laevis oocytes. J. Neurosci..

[72] Rasche S (2010). Tmem16b is specifically expressed in the cilia of olfactory sensory neurons. Chem. Senses.

[73] Corvol JC, Studler JM, Schon JS, Girault JA, Herve D (2001). Gα_olf_ is necessary for coupling D1 and A2a receptors to adenylyl cyclase in the striatum. J. Neurochem..

[74] Song Y (2008). Olfactory CNG channel desensitization by Ca^2+^/CaM via the B1b subunit affects response termination but not sensitivity to recurring stimulation. Neuron.

[75] Langenhan T, Sendtner M, Holtmann B, Carroll P, Asan E (2005). Ciliary neurotrophic factor-immunoreactivity in olfactory sensory neurons. Neuroscience.

[76] Asan E, Drenckhahn D (2005). Immunocytochemical characterization of two types of microvillar cells in rodent olfactory epithelium. Histochem. Cell Biol..

[77] Steinke A, Meier-Stiegen S, Drenckhahn D, Asan E (2008). Molecular composition of tight and adherens junctions in the rat olfactory epithelium and fila. Histochem. Cell Biol..

[78] Sambrook, J. & Russell, D. W. Molecular cloning – a laboratory manual (3^rd^ ed.) A8.45 (CSH Laboratory Press, Cold Spring Harbor, NY 2001).

[79] Mühlhans J, Brandstätter JH, Gießl A (2011). The centrosomal protein pericentrin identified at the basal body complex of the connecting cilium in mouse photoreceptors. PLoS One.

[80] Harazny J, Scholz M, Buder T, Lausen B, Kremers J (2009). Electrophysiological deficits in the retina of the DBA/2J mouse. Doc. Ophthalmol..

[81] Atorf J (2013). Functional protective effects of long-term memantine treatment in the DBA/2J mouse. Doc. Ophthalmol..

