# Supplementary material for: The BEACH Protein LRBA Promotes the Localization of the Heterotrimeric G-protein Golf to Olfactory Cilia
Source: Sci Rep. 2017 Aug 16;7:8409. doi: 10.1038/s41598-017-08543-4 (PMC5559528; doi:10.1038/s41598-017-08543-4)
Supplement: Supplementary file 1 — Supplementary Figures S1, S2 [file 41598_2017_8543_MOESM1_ESM.pdf]

Supplementary Figures

Title:

The BEACH Protein LRBA Promotes the Localization of the Heterotrimeric G-protein G_{olf} to Olfactory Cilia

Authors:

Stefan Kurtenbach, Andreas Gießl, Siv Strömberg, Jan Kremers, Jenny Atorf, Sebastian Rasche, Eva M. Neuhaus, Denis Hervé, Johann Helmut Brandstätter, Esther Asan, Hanns Hatt, and Manfred W. Kilimann

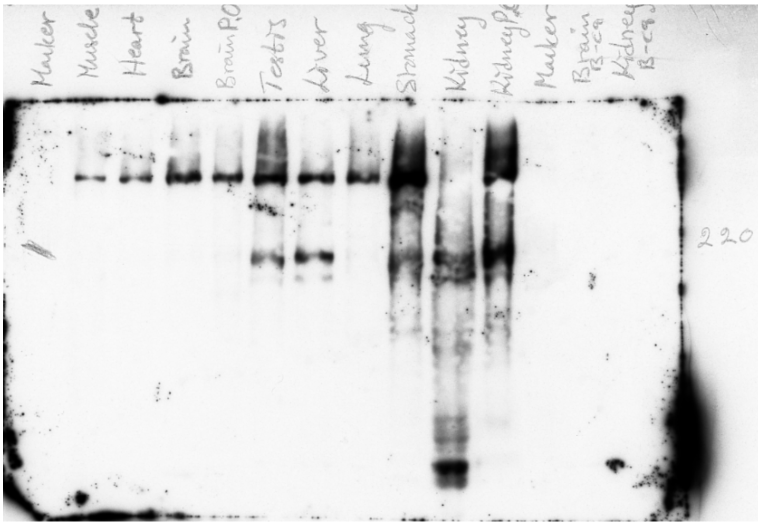

Supplementary Figure 1.

Unprocessed ECL film of the multi-tissue blot of Fig. 1A. Membrane top coincides with gel top; membrane bottom corresponds to 100 kDa; position of the 220 kDa marker is indicated on the right. Some tissues (testis, liver, stomach, kidney) displayed partial degradation (bands <220 kDa) of the large, 320 kDa LRBA protein. Kidney was repeatedly found particularly proteolysis-prone. All immunolabelled bands are abolished in the brain and kidney samples of LRBA-KO mice, on the right.

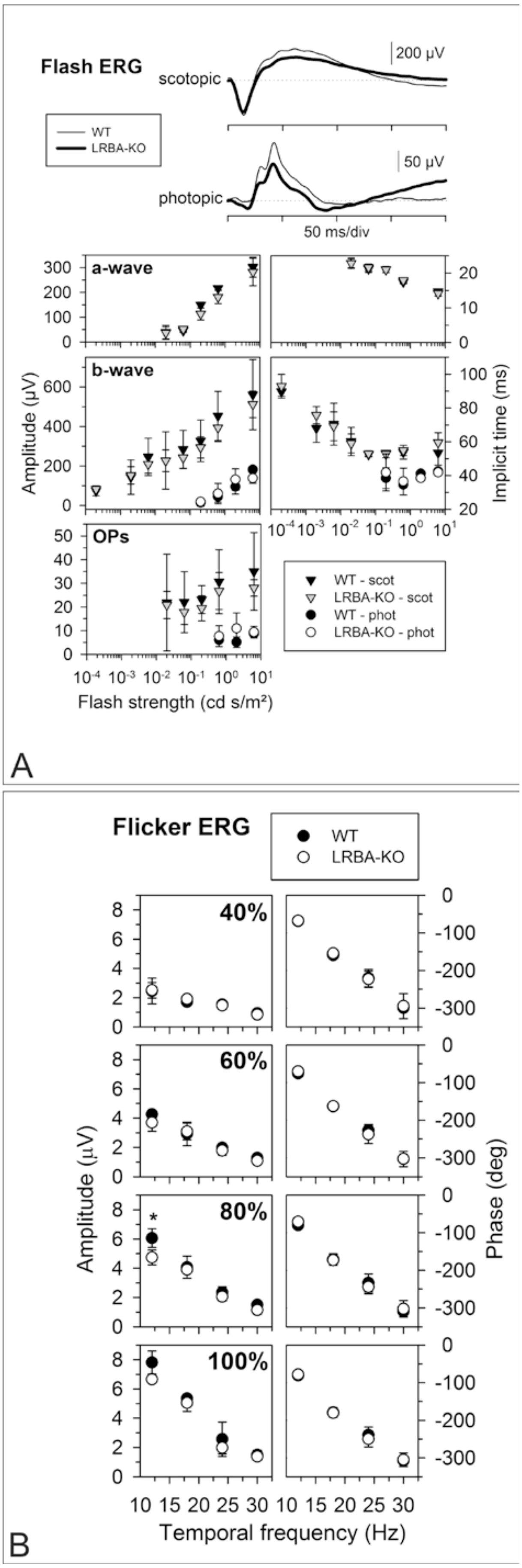

Supplementary Figure 2.

No electrophysiological dysfunction of the retina is detected in the LRBA-KO.

(A) Grouped average flash ERG waveforms (only signals to 6.3 cd s/m² shown), and corresponding parameters (amplitudes, left panels; implicit times, right panels) from WT (black symbols, n=2) and LRBA-KO mice (grey and white symbols, n=4), plotted as a function of flash strength (cd s/m²). Scotopic and photopic b-wave and OP peak-to-peak values are overlaid in the same panels (triangle and circular symbols, respectively). Note that parameters are shown only at relevant intensities.

(B) Fundamental component amplitudes (left panels) and phases (right panels) of flicker ERGs recorded with the different contrast modulations (40–100%, top to bottom panels) are given as a function of temporal frequency.
